# Orientation‐Confinement‐Engineered Stabilization of Ferroelectricity in HfO_2_ toward Maximum Polarization

**DOI:** 10.1002/advs.202521730

**Published:** 2026-03-02

**Authors:** Fatoye Sawyerr, Yongqing Sun, Zekun Zhang, Kang Jia, Shuning Lv, Qi Hu, Shu Shi, Qiushi Huang, Xie Zhang, Xiaoli Fan, Li‐min Liu, Shifeng Wen, Zheng Wen, Tengfei Cao, Jingsheng Chen

**Affiliations:** ^1^ Research Center for Advanced Lubrication and Sealing Materials School of Materials Science and Engineering Northwestern Polytechnical University Xi'an Shaanxi China; ^2^ College of Electronics and Information Shandong Key Laboratory of Micro‐nano Packaging and System Integration Qingdao University Qingdao China; ^3^ School of Physics Beihang University Beijing China; ^4^ Department of Materials Science and Engineering National University of Singapore Singapore Singapore; ^5^ Department of Materials Science and Engineering Northwestern Polytechnical University Xi'an China

**Keywords:** crystallographic orientations, electric polarizations, HfO_2_, oxygen vacancies, phase stabilities

## Abstract

Ferroelectric HfO_2_‐based materials are promising candidates for memory applications because of their compatibility with complementary metal‐oxide‐semiconductor (CMOS) technology. However, the ferroelectric phase of HfO_2_ is not the ground state, and collective displacements of oxygen atoms could generate multiple polarization switching paths, causing variations in measured polar magnitude across different experiments. To date, the mechanisms underpinning ferroelectric phase stabilization and the observed variations in polarization remain poorly understood. Here, by combining density functional theory (DFT) simulations and experimental measurements, we propose that (111) crystallography orientation confinement in Hf_0.5_Zr_0.5_O_2_ film can effectively stabilize the ferroelectric phase. Moreover, to account for different polarization magnitudes observed experimentally, we calculate the electric polarizations along different crystal orientations, incorporating both crossing and non‐crossing switching paths. These results show that all the crossing switching paths always yield high polarizations. However, relatively high switching barriers in crossing paths make them less likely to occur in measurements. Finally, to achieve high polarization together with low switching barriers, specific oxygen vacancies and cation dopants that facilitate crossing pathways and yield the highest polarization (∼70 µC/cm^2^) are determined. These insights clarify the preferred (111) orientation and polarization behavior of HfO_2_‐based films and advance the design of high‐performance ferroelectric devices.

## Graphical abstract

Ferroelectric phase stability in Hf₀._5_Zr₀._5_O_2_ films is enhanced by confinement to the (111) crystal orientation, as demonstrated by first‐principles calculations and experiments. Polarization approaching its maximum value is achieved through selective ferroelectric switching paths, which are further tuned by oxygen vacancies and cation doping to optimize ferroelectric performance.

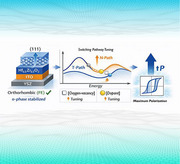

## 1 Introduction

The ferroelectric phase of HfO_2_ was first discovered in its silicon‐doped thin films [1], which immediately established HfO_2_ as a promising material for ferroelectric functions in integrated circuits. Because of its switchable electrical polarizations at the nanoscale and superior compatibility with current complementary metal oxide semiconductor (CMOS) technology, ferroelectric memories have been applied in a variety of memory devices, rekindling interest in creating nonvolatile, high‐speed, energy‐efficient storage devices [2]. Knowledge of ferroelectric physics, which was previously centered on perovskite oxides, has also been greatly impacted by the emergence of the ferroelectric phase in HfO_2_.

Ferroelectricity in hafnium is generally thought to originate from the formation of a metastable non‐centrosymmetric orthorhombic (*o*) phase with space group of Pca2_1_ [3, 4]. At ambient conditions, HfO_2_ is in a monoclinic (paraelectric) structure in the bulk phase. At 2773 and 1973 K, it transforms from the tetragonal phase (*t*‐phase, P4_2_/nmc) to the cubic phase (*c*‐phase, Fm3m) and finally to the monoclinic phase (*m*‐phase, P2_1_/c), respectively [5]. Unfortunately, all these phases are centrosymmetric and exhibit non‐polar properties. The polar phases assumed to be the most prevalent ferroelectric structures are the orthorhombic phase (Pca2_1_), rhombohedral (r3) phase, and another orthorhombic (Pmn2_1_) state, which are all high‐energy states with respect to the monoclinic states [3, 6, 7, 8, 9]. Several external factors have been proposed to explain the stabilization of ferroelectric phases in thin films [10, 11], including cation or anion doping, defect engineering, as well as kinetic mechanisms, and so on. All these factors have been identified as crucial factors for stabilizing the metastable polar phase in the preparation processes in different experiment groups [3, 12]. Interestingly, thin‐film epitaxy is an effective technique for comprehending the ferroelectric behavior of HfO_2_, because of its well‐regulated substrate‐ferroelectric interactions and microstructures [6]. The phase stability of HfO_2_ polymorphs can be controlled for ferroelectric characteristics and device prototyping by substrate symmetry and properties, as well as the lattice mismatch between the film and substrate [13]. It has been demonstrated that isotropic substrates with a favorable lattice constant can stabilize the Pca2_1_ phase within a broad range of lattice parameters (5.00 Å–5.23 Å) [7], beyond which the *m*‐phase exhibits higher stability. Under high compressive strains, both r3 and Pca2_1_ become competitive phases, however, the nonpolar *m*‐phase is still the most stable under these epitaxial circumstances [2, 14]. According to Fina and Sánchez [15, 16], there is a significant controversy between DFT calculations and experimental measurements on the necessary strain contributing to polarization when the r3 phase is assumed in (111)‐oriented HZO films [17].

Additionally, the ferroelectric switching processes in HfO_2_ are fundamentally different from those in perovskites (ABO_3_). In ABO_3_, polarization switching is governed by the off‐center displacements of B‐site cations, which yield a fixed and well‐defined switching path [18, 19]. In HfO_2_ films, polarization switching arises from connective displacements of oxygen atoms. Polarization kinetics in the *o*‐phase of HfO_2_ have been studied, including strain and doping effects [20, 21]. However, little is known about how polarization switching occurs under real measurement conditions across diverse samples and experimental settings. High spontaneous polarization (*Ps*) in HfO_2_‐based films is needed to enhance ferroelectric device performances, particularly reliability and data retention [6]. Achieving the highest *Ps* values near the theoretical maximum in HfO_2_‐based films remains experimentally challenging, suggesting that particular switching pathways should be selectively stabilized among the available options. Thus, substantial experimental and theoretical debates remain on the most effective phase‐stabilization techniques and on the mechanisms of ferroelectric polarization switching [2].

In this study, we first use DFT simulations to analyze (111) crystal orientation confinement effects on phase stabilities of pure HfO_2_ and (Hf_0.5_Zr_0.5_O_2_) HZO films under varying epitaxial strain across different substrates, demonstrating that the ferroelectric phase can be efficiently stabilized. Moreover, we carry out extensive experiments by epitaxially growing Hf_0.5_Zr_0.5_O_2_ thin films along the (111) and (001) directions. The experimental results demonstrate that the (111) confined orientation can proficiently stabilize the ferroelectric phase. To rationalize different polarization magnitudes observed experimentally, we calculate the electric polarization of HfO_2_ films along different crystal orientations, considering both crossing and non‐crossing switching paths. All crossing paths yield higher polarizations than the corresponding non‐crossing ones, but they also involve higher energy barriers, reducing their likelihood of occurrence in measurements. Finally, to achieve high polarization with a preferred switching pathway, we analyze the effects of both oxygen vacancies and cation doping on crossing and non‐crossing paths. We identify various oxygen vacancies that favor crossing paths and produce high polarization values. Among cation dopants, strontium, samarium, and europium are all predicted to increase the likelihood of crossing paths and thereby enhance the measured high polarization in HfO_2_ films.

## 2 Results and Discussion

### 2.1 (111) Crystal Orientation Effects on HfO_2_ Phase Stabilities

**FIGURE 1:**
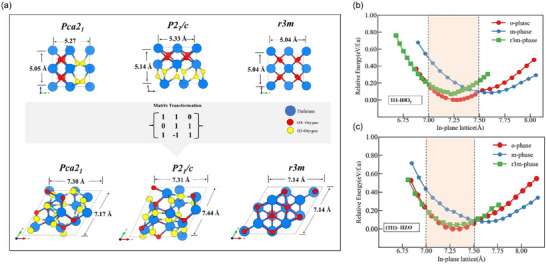
(111)‐Orientation Confinement and Phase Stabilities: (a) The structure, lattice parameters, and space group of the *o*‐, *m*‐, and *r3m*‐phases before applying matrix transformation. The transformed three phases (*o, m, r3m*) into (111) orientation exhibiting (111) surface. The O_3_ (O_4_)‐oxygen indicates oxygen atoms forming three (four) metal‐oxygen bonds with surrounding Hf atoms. (b, c) Computational results on epitaxial stability of pure HfO_2_ and HZO polymorphs (*o‐*phase, *m*‐phase, *r3m* phase).

Lots of experimental results have indicated that Hf_0.5_Zr_0.5_O_2_ films grown on perovskite substrates with the conductive LaSrMnO_3_ buffer layer always prefer the (111) orientation [22]. Here, we first examine Hf_0.5_Zr_0.5_O_2_ structures with the (111) orientation in the monoclinic (*m*‐phase), rhombohedral (*r3m*‐phase), and orthorhombic (*o*‐phase) phases. In the *r3m*‐phase, the (001) surface of its crystal unit cell is equivalent to the (111) surface of the primitive cell, which is used here to compare this with both the *o*‐phase and *m*‐phase. In Figure 1, we show the transformation matrix that is applied to the unit cells of the *o*‐, *m*‐, and *r3m*‐phases to reveal their (111) crystallographic orientations. By applying this matrix to the unit cell of *o*‐ and *m*‐phases, as well as the primitive cell of *r3m*‐phase, we find that all three structures can be transformed into similar hexagonal‐like supercells containing 12 Hf atoms and 24 O atoms. For clarity, the lattice parameters of all unit cells and the transformed supercells are provided in Figure 1. The space group for each phase with (111) orientation along the out‐of‐plane direction is also provided in the corresponding structures. It can be observed in Figure 1a that, for all three above phases that grow along the (111) directions, there are crystal orientation confinements on them. Moreover, these three phases are energetically competitive under in‐plane epitaxial strain, and constitute the main focus of the calculations presented here.

HfO_2_ films grown in the (111) orientation may experience either tensile or compressive strain because of lattice mismatches with different substrates. This epitaxial strain can influence the relative stabilities of the different crystal phases. To identify the possible stabilization effects of confinement on the ferroelectric *o*‐phase, we treat the in‐plane lattice parameters as variable inputs and evaluate how epitaxial strain impacts the stabilities of different phases. Moreover, oxygen vacancies are almost inevitable defects in the HfO_2_ matrix, and their existence could also impact the relative stabilities of different phases. Therefore, oxygen vacancy effects are also included during the ferroelectric phase stabilization analysis.

Figure 1b,c shows energy variations of (111)‐oriented pure HfO_2_ and Hf_0.5_Zr_0.5_O_2_ polymorphs (*o*‐phase, *m*‐phase, *r3m*‐phase) with respect to in‐plane lattice (Å) (other concentration ratios were also calculated (see Figure S1)). Here, our calculation shows that the r3m phase has a lower energy when the lattice parameter is between 6.75 Å and 7.0 Å. This means the rhombohedral phase becomes more stable if in‐plane compressive strain is applied to the film. The strain quantification is provided for analysis in Table S1. At an in‐plane lattice of 7.26 Å, the *o*‐phase energy reaches the global minimum, thus indicating that compressive and tensile strain (‐1% to 3%) keeps the orthorhombic ferroelectric phase stable. Beyond lattice parameters of 7.5 Å, the *m*‐phase gains stability as its energy becomes the lowest one.

The existence of oxygen vacancies in the HfO_2_ matrix could also impact confinement effects on phase stabilities of *o*‐, *m*‐, and *r3m*‐HfO_2_. Our results show that, at 4.2% and 8.3% oxygen vacancy concentrations, the energy minimum of the *o*‐phase remains at 7.25 Å, and slight compression or tensile strain is needed to stabilize the *o*‐phase over the *m*‐phase (see Figure S1a) in HfO_2_ films containing oxygen vacancies. Unlike (111)‐oriented structures, films grown along the (001) and (110) directions are less favorable, because the *m*‐phase is energetically more stable than the *o*‐phase over a wide range of epitaxial strain, and rather large compressive strains are needed to stabilize the *o*‐phase (Figure S1c) [1, 23].

We further calculated the phase‐transition pathway and the energy barrier between the *o*‐ and the *m*‐phase under (111) crystal orientation confinement (Figure S1e). The lattice parameter corresponding to the lowest energy *o*‐phase is used for both phases (a = b = 7.25 Å) here. The results show that the *m*‐phase can transform into the *o*‐phase by crossing a rather low energy barrier of 0.14 eV per unit cell, promoting *o*‐phase formation during the epitaxial growth of HfO_2_ films.

Figure 1b,c demonstrates that, for in‐plane lattice parameters within the range from 7.0 Å to 7.5 Å, ferroelectric *o*‐phase can be stabilized. According to these lattice parameters within this range, we further examined the Material Project database [24], and identified several materials with favorable lattice constants, including Y_2_O_3_, In_2_O_3_, LaSrMnO_3_, SrTiO_3_, LaAlO_3_, Gd_2_O_3_, Sc_2_O_3_, and others (See Table S2). Among these, LaSrMnO_3,_ SrTiO_3,_ and LaAlO_3_ are commonly employed as substrates, while some of the others can also serve as potential bottom electrode materials depending on the device architecture. Since the r3 confined orthorhombic phase of HfO_2_ (111) surface has a triangular in‐plane lattice approximately 7–7.5 Å, the optimum substrates are {111} surface for metal oxides, (001) perovskite with this lattice parameter window [17]. Specifically, the substrates shown in Table S2 provide favorable growth directions within the desired window with mismatches ≤ ≈ 7% [17].

**FIGURE 2:**
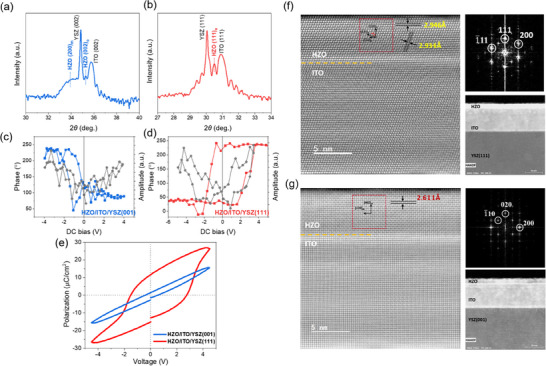
Experimental results on the phase and ferroelectric properties of the HZO thin films grown on (001)‐ and (111)‐oriented YSZ substrates. (a, b) XRD *θ*‐2*θ* scans, (c, d) PFM phase and amplitude loops, and (e) *P–V* hysteresis loops of the HZO thin‐film capacitors measured at 10 kHz. (f, g) HAADF‐STEM image of the HZO/ITO/YSZ 5 nm thin‐film heterostructures, magnified HAADF‐STEM image and corresponding fast fourier transform (FFT) pattern of the HZO (111) and HZO (001) layer indicated by the dashed red box, for phase analysis.

The theoretical calculations are verified by performing comparative experiments on the Hf_0.5_Zr_0.5_O_2_ (HZO) thin films grown on (001)‐ and (111)‐oriented YSZ substrates, respectively, buffered with conductive indium tin oxide (ITO) as the bottom electrodes. Due to small lattice misfits among the HZO, ITO, and YSZ, we achieved the {100} and {111} family of crystal planes in the epitaxially‐textured HZO thin films (Figure S2). Specifically, on the (001)‐oriented YSZ, the HZO exhibits the coexistence of (200)_m_ and (002)_o_ orientations (Figure 2a), while the non‐polar *m*‐phase is suppressed on the (111)‐oriented YSZ, in which only the (111)_o_ orientation is observed in the HZO thin film (Figure 2b). Ferroelectric properties of the HZO/ITO/YSZ heterostructures are characterized by means of piezo‐response force microscopy (PFM). As shown in Figure 2c), there is almost no ferroelectric hysteresis behavior observed in both the phase and amplitude loops, corresponding to the *m*‐phase domination of the HZO/ITO/YSZ (001) (Figure 2a). For the HZO/ITO/YSZ (111), a 180° phase contrast and butterfly‐type amplitude hysteresis are observed in the PFM loops, suggesting robust ferroelectricity owing to the stabilization of the *o*‐phase (Figure 2b). The difference in ferroelectric behaviors is also shown in PFM phase and amplitude images (Figure S3). Furthermore, by employing Pt as the top electrodes, the Pt/HZO/ITO thin film capacitors are fabricated for measuring polarization–voltage (*P–V*) hysteresis loops by the conventional Sawyer‐Tower method. It is clear that improved ferroelectric properties are achieved in the HZO thin film on (111)‐oriented YSZ substrate, showing the *Ps* of 28 µC/cm^2^ and remanent polarization of ∼12 µC/cm^2^ before the electrical breakdown of the capacitor. The ferroelectric switching is also confirmed by the switching currents, in which two current peaks appear at the coercive voltages (Figure S2b).

High‐angle annular dark‐field scanning transmission electron microscopy (HAADF‐STEM) was employed to directly identify the crystalline phases present in the HZO layer. Figure 2f,g show representative HAADF‐STEM images of the orthorhombic *o*‐phase region oriented along the (111) direction and the monoclinic *m*‐phase region oriented along the (002) direction, respectively. In Figure 2f, the measured lattice spacing of the (111) plane is 2.946 Å, as indicated by the black guide lines, and the corresponding fast Fourier transform (FFT) pattern agrees well with previously reported values for *o*‐phase HZO in the (111) orientation, confirming the phase assignment. Similarly, Figure 2g shows a lattice spacing of 2.611 Å for the (002) planes of the *m*‐phase region, and its FFT pattern is also consistent with prior literature reports [22, 25]. These observations unambiguously confirm the coexistence of *o*‐phase (111) and *m*‐phase (002) domains within the HZO layer. This experimentally observed lattice spacing represents the interplanar distance of the (111) plane, which is crystallographically linked to the projected in‐plane periodicity imposed in the orientation‐confined (111) theoretical model. Consequently, the experimentally measured values correspond to the same in‐plane strain regime explored in our calculations and are fully consistent with our simulation findings that confinement along the (111) crystal orientation facilitates stabilization of the ferroelectric *o*‐phase.

### 2.2 Electronic Polarization Along Different Crystal Orientations

**FIGURE 3:**
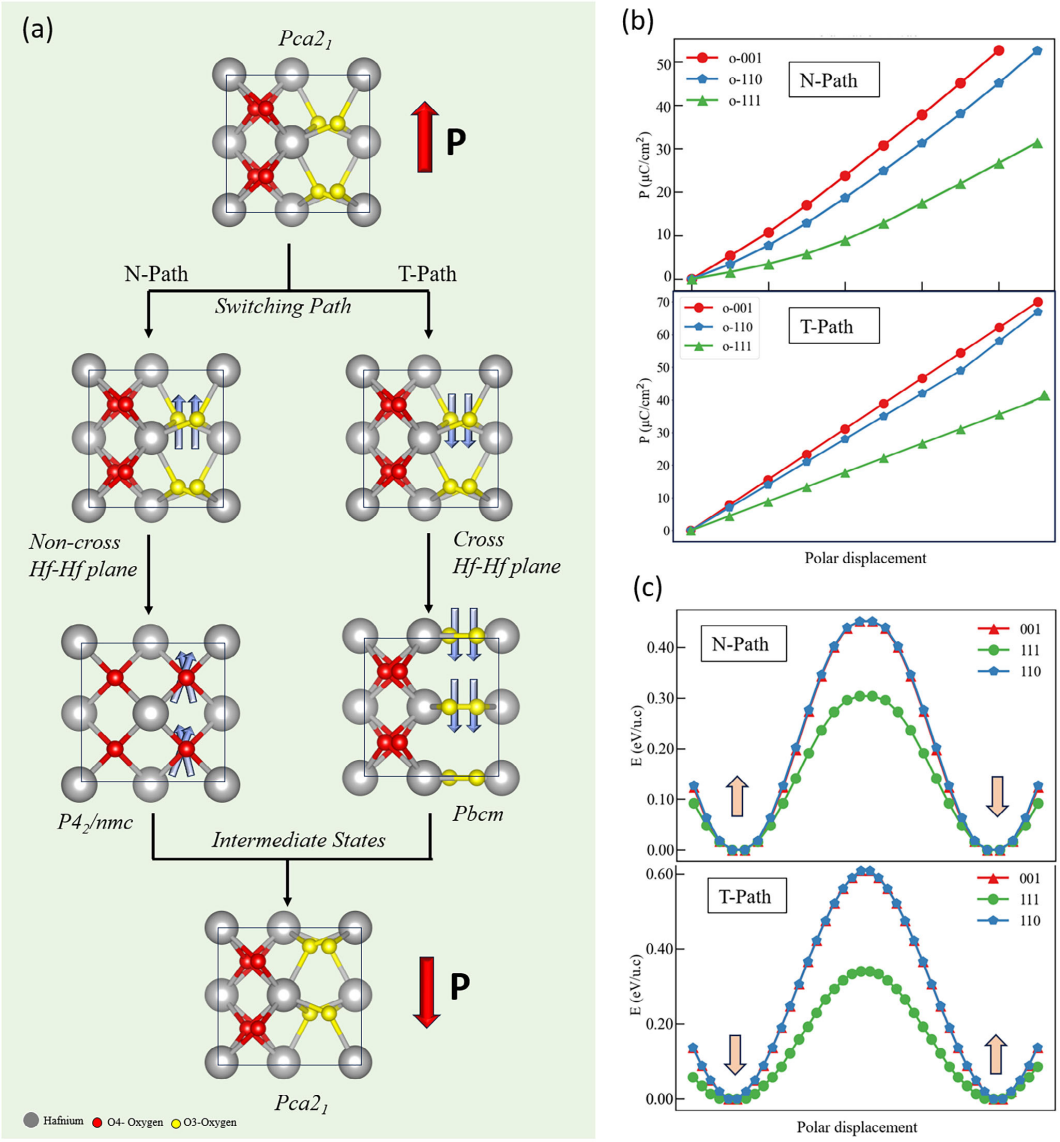
Polarization of ferroelectric *o*‐phase for both N‐ and T‐path (a) The N‐ and T‐path of ferroelectric switching in HfO_2_ matrix. (b) Polarization magnitude of HfO_2_ polymorphs in the (001), (110), and (111) oriented directions for both N‐ and T‐path. (c) Ferroelectric double‐well energy barrier of the ferroelectric switching processes for both N‐ and T‐path of HfO_2_ along (001), (110), (111) oriented directions.

The polarization switching in HfO_2_ is caused by the collective displacement of oxygen atoms with relatively small atomic radius, which gives them more mobility inside the HfO_2_ matrix and greatly increases the variety and complexity of polarization reversal events in HfO_2_. Recently, it was argued that polarization switching can be divided into two main categories depending on the displacement direction of threefold coordinated O atoms passing through (T‐path) or not through (N‐path) the Hf‐Hf atomic planes [26, 27], and leads to two distinct polarization magnitudes. We illustrate the different polarization switching paths in Figure 3a. Accordingly, we also calculate the electric polarizations of HfO_2_ films along different crystal orientations, including both the T‐ and N‐path. As it is shown in Figure 3b, for the N‐path, the electric polarizations along (001), (110), and (111) are calculated to 51.3 µC/cm^2^, 53.8 µC/cm^2^, and 30.2 µC/cm^2^, respectively. Correspondingly, for the T‐path, the electric polarizations are 69.9 µC/cm^2^ (001), 69.5 µC/cm^2^ (110), and 40.3 µC/cm^2^ (111), respectively. All these results demonstrate that, unlike conventional perovskite materials, HfO_2_ films exhibit multivalued polarization characteristics. This multivalued behavior first manifests in different crystallographic orientations, where the polarization magnitudes vary. For example, in the N‐process, the polarization along the (111) direction is 30.2 µC/cm^2^, whereas along the (001) direction it is 51.3 µC/cm^2^. Consequently, because of different growth conditions, the synthesized HfO_2_ thin films may exhibit different crystallographic orientations and, accordingly, different polarization magnitudes. More importantly, even for the same crystallographic orientation, whether oxygen atoms cross the Hf–Hf atomic layer during polarization switching or not can lead to markedly different polarization strengths (e.g., 51.3 vs 69.9 µC/cm^2^ along the (001) direction). These findings partly explain the multivalued polarization strengths across different experiments [10, 28, 17, 29].

In Figure 3c, we calculated the ferroelectric double‐well energy barriers associated with polarization reversal along different crystallographic orientations, extracted from symmetry‐constrained switching pathways connecting the two stable polarization states [51]. These results show that, for the N‐path, the energy barriers of the ferroelectric polarization switching in (001), (110), and (111) directions are 0.46 eV/u.c, 0.47 eV/u.c, 0.30 eV/u.c, respectively. For the T‐path, the barriers are 0.60 eV/u.c. (001), 0.61 eV/u.c. (110), and 0.33 eV/u.c. (111), correspondingly. A similar trend is seen in the VC‐NEB calculated switching energy barriers for the N‐ and T‐paths in Figure S7, which corresponds to the symmetry‐allowed polar axis and the experimentally relevant out‐of‐plane orientation in thin films, showing that the T‐path exhibits a higher barrier (0.095 eV/f.u.) than the N‐path (0.056 eV/f.u.). This difference originates from the distinct intermediate states and lattice distortion modes involved during polarization reversal (Figure S6). Specifically, during 180° polarization switching, the non‐crossing (N‐path) pathway proceeds via a tetragonal intermediate phase (P4_2_/nmc), with a distortion pattern that lowers the overall energy barrier. Phonon analysis reveals that the P4_2_/nmc phase possesses three unstable modes ($\Gamma_{5}^{-}$, *M*_1_, and *M*_3_) that strongly couple to and facilitate stabilization of the polar Pca2₁ phase, thereby promoting an energetically favorable switching process. In contrast, the crossing (T‐path) pathway involves a Pbcm intermediate phase with a higher associated energy barrier; notably, this phase exhibits only a single unstable mode ($\Gamma_{2}^{-}$) that couples to the polar Pca2₁ phase, resulting in reduced lattice instability and a less efficient switching pathway [21, 30]. More generally, these results demonstrate that the T‐path consistently exhibits higher polarization‐switching barriers, which can be attributed to the substantial structural deformations induced when oxygen atoms cross the Hf–Hf plane during switching, making this pathway energetically less favorable and therefore less likely to occur experimentally.

### 2.3 Oxygen Vacancy Effect On N‐ And T‐Paths

The above results demonstrate that the N‐ and T‐paths are important polarization switching processes in HfO_2_. Although the T‐paths involve relatively high energy barriers, they can also yield large electric polarizations, which are valuable for data storage. Oxygen vacancies are dominant defects in HfO_2_ thin films. The presence of different oxygen‐vacancy configurations can selectively favor the N‐ or T‐path, thereby influencing the measured ferroelectric polarizations. Here, we further analyze how oxygen vacancies affect the N‐ and T‐paths in HfO_2_. For simplicity, we consider an oxygen‐vacancy concentration of 8.3%, corresponding to introducing two oxygen vacancies into the 36‐atom supercells (Figure 1a). Moreover, these relative positions of two oxygen vacancies can yield distinct vacancy configurations, allowing us to explore how oxygen conformations influence both the N‐ and T‐paths. Here, we investigated all possible 2‐oxygen vacancy (8.3%) configurations of our 36‐atom HfO_2_ *o*‐phase supercell, and the results in a total of 276 different structures (see Figures S11 and S12) [31].

**FIGURE 4:**
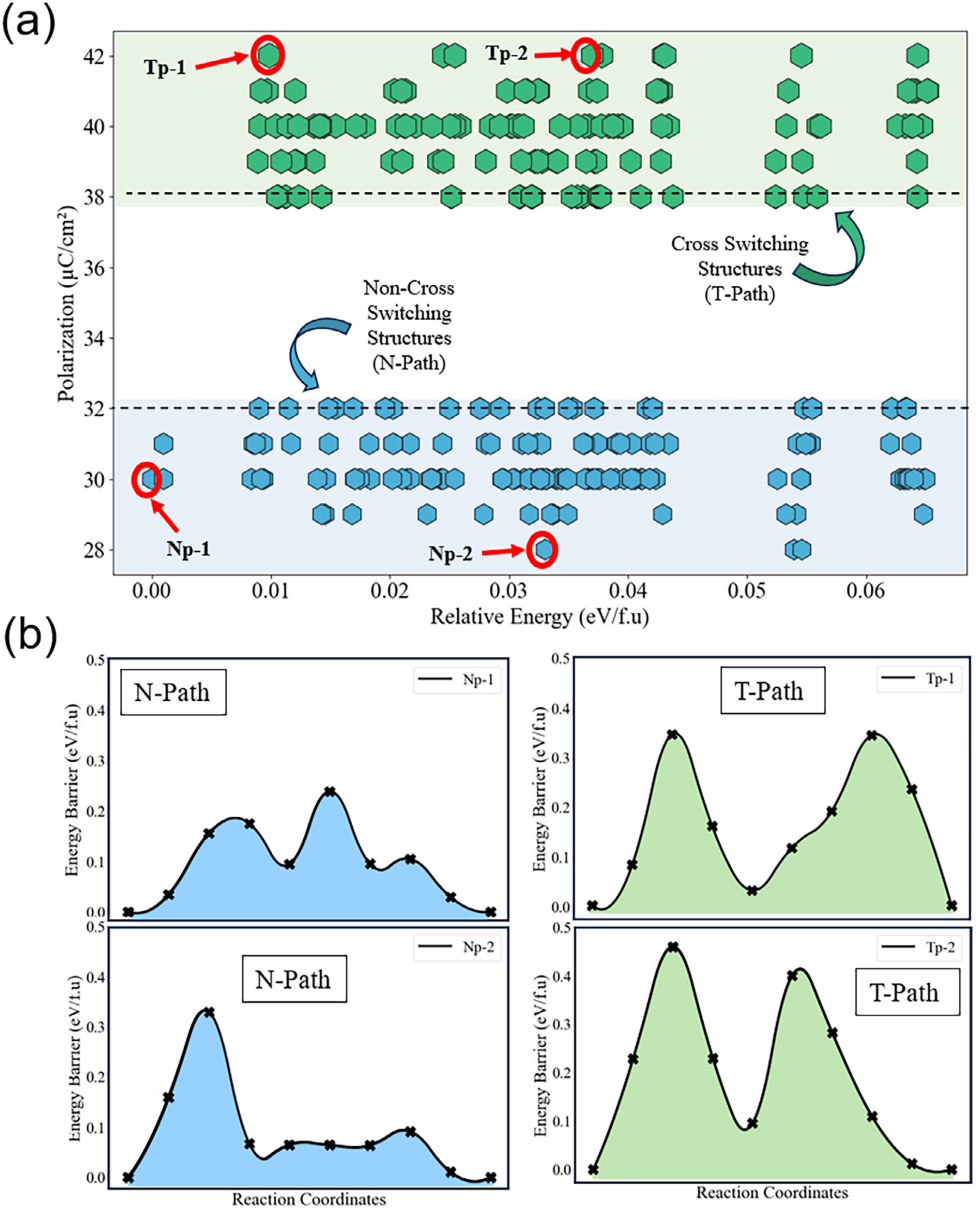
Energy barrier and polarization of (111) ferroelectric HfO_2_ with oxygen vacancy (a) Polarization and relative energies of 8.3% oxygen vacancy structures generated by creating all 276 vacancy site variations. (b) Switching energy barrier of N‐path and T‐path for selected oxygen vacancy configurations; Np‐1, Np‐2, and Tp‐1, Tp‐2, respectively.

We calculated the total energy of each structure, and all related results are given in Figure 4a, with the energy of the most stable structure used as the reference. The largest energy difference among the different two‐oxygen‐vacancy configurations is 0.065 eV per formula unit (0.065 eV/f.u.). It means that although these two‐oxygen‐vacancy configurations can have different formation energies, the energy differences between the two‐oxygen‐vacancy configurations are relatively small. Therefore, all these two‐oxygen‐vacancy configurations could exist in the HfO_2_ matrix under different growing conditions [32].

We further compute the electric polarization of HfO_2_ films to assess whether the presence of two‐oxygen‐vacancy configurations biases toward the N‐path or T‐path, thereby influencing the final polarization. All results in Figure 4a clearly show that these two oxygen vacancies could select either the T‐path or N‐path, depending on their configurations. The highest polarization values (38–42 µC cm^2^) in (111) orientation of HfO_2_ films occur for the T‐path, with the corresponding two‐oxygen vacancies depicted in Figure 4a. Polarization values in the range 28–32 µC cm^2^ correspond to the N‐path and are associated with the other two oxygen vacancies. These results specifically demonstrate that oxygen vacancies in HfO_2_ thin films exhibit selective polarization reversal for the N‐path and the T‐path. For either path, the associated oxygen vacancies can be distributed across the entire energy range, and the total number of vacancy types is roughly similar. To further understand the switching mechanism for the N‐ and T‐paths, we calculate the switching energy barrier of selected oxygen vacancy structures for the N‐ and T‐paths as shown in Figure 4b, using their energies as reference. The results show N‐path structures with energy 0.00 eV/f.u. (Np‐1) and 0.032 eV/f.u. (Np‐2) having switching energy barriers of 0.24 eV/f.u. and 0.33 eV/f.u., respectively. Whereas, the T‐path structures with energy 0.01 eV/f.u. (Tp‐1) and 0.038 eV/f.u. (Tp‐2) have a higher switching energy barrier of 0.36 eV/f.u. and 0.46 eV/f.u. respectively.

This evidently explains why the N‐path is likely to occur during experiments due to the low switching barrier. These results also indicate that while oxygen vacancies do not universally stabilize the T‐path, certain vacancy configurations can locally favor T‐path‐like switching, with the small energy difference to the N‐path (∼0.01 eV/f.u.) indicating close energetic competition driven by defect‐induced symmetry breaking. Consequently, in practically grown samples, it is difficult to identify which type of oxygen vacancy is present, contributing to variability in polarization magnitude observed across samples and between different experimental groups [10, 26, 28, 33, 29].

### 2.4 Doping Effect on Polarization Switching

**FIGURE 5:**
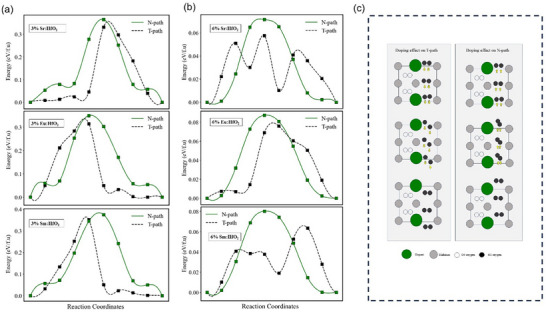
Cation Doping Effects on Switching Barriers of N‐path and T‐path for Ferroelectric HfO_2_. Polarization switching (polar up to polar down) energy profile for doped HfO_2_ N‐path and T‐path with (a) 3.125% and (b) 6.250% concentration of Sr, Eu, and Sm dopants. (c) Schematic structure deformations in cation‐doped HfO_2_ for N‐path and T‐path.

In addition to oxygen vacancies, cation doping is another common strategy to modulate the stabilities and electric polarizations of HfO_2_ films [34, 35]. Here, we further examine how the cation doping impacts the relative barriers between the T‐path and N‐path. Thus, we considered cation‐doping concentrations of 3.125% (96 atoms), 6.250% (48 atoms), and 25% (12 atoms). However, our discussion here focuses on the lower doping levels, as these correspond to experimentally realistic conditions. Importantly, all concentration results reproduce the same qualitative trend observed, indicating dopant incorporation selectively favors the switching barrier along the T‐path over the N‐path. This demonstrates that the pathway‐selective effect of cation doping is robust and relevant to experimentally accessible regimes. The corresponding results are shown in Figure 5. The doping of Sr, Eu, and Sm all contribute to the selection of ferroelectric switching of the T‐path. For example, in the 3.125% and 6.250% Sr‐doped case in Figure 5a,b, the T‐path polarization‐reversal barrier is 0.33 eV/f.u., 0.057 eV/f.u., respectively, while the N‐path barrier is 0.36 eV/f.u., 0.07 eV/f.u., respectively. The barrier difference indicates that the T‐path has a lower barrier; it is likely to be dominant during experiments. In Eu‐ and Sm‐doped cases, the T‐path and N‐path polarization‐reversal barriers demonstrate a similar trend for 3.125% and 6.250% concentrations, where the T‐path is favored over the N‐path. The energy barrier for 3.125% and 6.250% concentration for T‐path is 0.329 eV/f.u. and 0.075 eV/f.u., respectively, for Eu, and 0.342 eV/f.u. and 0.082 eV/f.u. respectively for Sm (Figure 5a,b). In contrast to pristine HfO_2_, dopant incorporation can increase the polarization switching barrier in HfO_2_ by locally distorting the Hf‐O coordination and breaking coherent oxygen‐sublattice motion, forming dopant‐defect complexes that pin the lattice at the transition state, and hardening lattice modes involved in polarization reversal, thereby raising the NEB saddle‐point energy despite stabilization of the polar phase [36, 37, 29, 35]. Thus, Sr, Sm, and Eu doping yield a T‐path barrier that is lower than that of the N‐path.

All these results can be understood in terms of lattice variations of the HfO_2_ matrix during polarization reversal [33]. For example, Sr doping induces larger lattice distortions during N‐path switching processes; this distortion moves the O3‐oxygen atoms closer, affecting the conventional upward motion and causing an increase in the formation (see Figure S9). Whereas, during T‐path switching, distortion from doping moves the O_3_‐oxygen atoms away from each other, therefore allowing the atoms to cross the Hf‐Hf plane one by one, unlike pure HfO_2_ switching, where both atoms cross together. This reduces the energy cost for the structure deformation, resulting in a smaller T‐path barrier as illustrated in Figure 5c.

## 3 Conclusion

In this study, we combine density functional theory (DFT) simulations with experimental measurements to show that confinement to the (111) crystal orientation can effectively stabilize the ferroelectric phase in HZO films. Theoretical calculations indicate that the (111) film orientation facilitates the stabilization of the HZO ferroelectric phase. XRD and HAADF‐STEM image patterns and ferroelectric characterizations show that HZO films grown along the (111) orientation on ITO/YSZ substrates are dominated by the orthorhombic phase and exhibit robust ferroelectric properties; by contrast, (001)‐oriented HZO films display reduced ferroelectric polarizations due to the competition of monoclinic phases. In the ferroelectric phase of HfO_2_, polarization associated with crossing switching paths (T‐path) surpasses that of non‐crossing paths (N‐path) across various crystal orientations. Nevertheless, T‐paths generally incur higher energy barriers, reducing their occurrence in experiments. We further examine how oxygen vacancies and cation doping influence the T‐path and N‐path to achieve high polarization. The effect of oxygen vacancies depends on their configuration; they can promote either the T‐path or the N‐path, leading to higher or lower polarizations observed experimentally. Conversely, dopants such as Sr, Eu, and Sm increase the likelihood of T‐paths, thereby enhancing the maximum polarization measured in HfO_2_ films. Collectively, these findings indicate that engineering epitaxial strain, oxygen vacancies, and cation doping can substantially improve the ferroelectric properties of HfO_2_ films for memory devices and related electronic systems.

## 4 Experimental Section/Methods

### 4.1 Computational Method

All density functional theory calculations are carried out by applying the Vienna ab initio simulation package (VASP) [38]. The Perdew‐Burke‐Ernzerhof (PBE) exchange correlation functional is used to describe the electron exchange‐correlation interactions [39], and the projector augmented‐wave (PAW) method accounts for the electron‐ion interactions [40, 41]. For all calculations, the 500 eV is chosen for the plane‐wave energy cutoff. The Γ‐centered 4 × 4 × 4 K‐point mesh for the crystal unit‐cell and 3 × 2 × 3 for the 1 × 2 × 1 supercell, are utilized within the Monkhorst‐Pack method [42]. The switching energy barriers are all calculated using the generalized solid‐state variable cell nudged elastic band (VC‐NEB) method [43]. Dopant calculations were performed using 2 × 2 × 2 and 2 × 2 × 1 supercells (96 and 48 atoms in total) to introduce 3.125% and 6.25% doping concentrations. The ferroelectric polarizations of HfO_2_ are calculated according to the Berry phase method [19]. In‐plane epitaxial strain is imposed by adjusting the lattice parameters to predefined values and fixing them at those values for HfO_2_ films oriented along the (111) direction, with both atomic coordinates and the out‐of‐plane lattice parameter fully optimized [44, 45]. The energy of various phases was calculated with respect to the most stable ferroelectric orthorhombic HfO_2_. The following equation is used to determine the relative energy (eV/f.u) of a phase with respect to the number of cation atoms, Δ*E*:

$$\Delta E=\frac{E_{s}-E_{m}}{n_{c}}$$

*E_s_* is the energy of all structures under different epitaxial strain. *E_m_* is the minimum strain energy of all calculated structures in the whole energy profile *n_c_* is the total number of formulas in the system, accordingly. The Atomic Simulation Environment (ASE) and itertools Python packages are used to generate different oxygen vacancy configurations in HfO_2_ matrix [46, 47].

### 4.2 Sample Preparations and Characterizations

Hf_0.5_Zr_0.5_O_2_ and ITO thin films were deposited on (111)‐ and (001)‐oriented YSZ substrates by pulsed laser deposition using a KrF excimer laser. The ITO, about 20 nm in thickness, was deposited at a laser energy density of ∼2.8 J/cm^2^ with a repetition rate of 5 Hz, keeping the oxygen pressure at 0.02 mbar [25, 48]. The Hf_0.5_Zr_0.5_O_2_ were deposited at a laser energy density of ∼2 J/cm^2^ with a repetition rate of 4 Hz, keeping the substrate at 650°C and the oxygen pressure at 0.1 mbar [49]. The thin‐film thickness was controlled by counting the laser pulse number and measured by atomic force microscopy, associated with a designed step during the growth [50]. Pt top electrodes of ∼30 µm in diameter and ∼50 nm in thickness were deposited by a DC magnetron sputtering through a shadow mask for the fabrication of thin‐film capacitors. XRD was performed on a Rigaku Smart Lab X‐ray diffractometer with Cu Kα radiation. Ferroelectric hysteresis loops were measured by a Radiant Premier II ferroelectric tester at a frequency of 10 kHz. High‐angle annular dark‐field scanning transmission electron microscopy (HAADF‐STEM) was performed at an accelerating voltage of 200 kV using a spherical‐aberration‐corrected scanning transmission electron microscope (FEI Titan Themis 200). Cross‐sectional TEM specimens were prepared by focused ion beam (FIB) milling using a Ga^+^ ion source (FEI Versa workstation). Additional HAADF‐STEM imaging was conducted at 200 kV on a JEOL ARM200CF microscope equipped with a cold field‐emission electron gun, an ASCOR probe aberration corrector, and a Gatan Quantum ER spectrometer [52].

## Funding

National Natural Science Foundation of China (Program Nos. 12474061 and 52372113), Youth Project of Shanxi High‐level Talents Introduction Plan (5113240032), the Innovation Capability Support Program of Shaanxi (Program No. 2024RS‐CXTD‐30), the Advanced Materials‐National Science and Technology Major Project (2026ZD0623501), Taishan Scholar Program of Shandong Province (Grant No. tstp20240511), Shaanxi Qinchuangyuan High‐Level Innovative and Entrepreneurial Talent Introduction Program (QCYRCXM‐2023‐077).

## Conflicts of Interest

The authors declare no conflict of interest.

## Supporting information




**Supporting File**: advs74484‐sup‐0001‐SuppMat.docx.

## Data Availability

The data that support the findings of this study are available in the supplementary material of this article.
