# Supplementary material for: Orientation‐Confinement‐Engineered Stabilization of Ferroelectricity in HfO2 toward Maximum Polarization
Source: Adv Sci (Weinh). 2026 Mar 2;13(24):e21730. doi: 10.1002/advs.202521730 (PMC13116380; doi:10.1002/advs.202521730)
Supplement: Supplementary file 1 — Supporting File: advs74484‐sup‐0001‐SuppMat.docx. [file ADVS-13-e21730-s001.docx]

**Supplementary Information**

**Orientation-Confinement-Engineered Stabilization of Ferroelectric Phases in HfO_2_ for Maximum Polarization**

*Fatoye Sawyerr, Yongqing Sun, Zekun Zhang, Kang Jia, Shuning Lv, Qi Hu, Shu Shi, Qiushi Huang, Xie Zhang, Xiaoli Fan, Li-min Liu, Shifeng Wen^*^, Zheng Wen^*^, Tengfei Cao^*^，Jingsheng Chen*

F. Sawyerr, Z. Zhang, K. Jia, X. Fan, T. Cao

Research Center for Advanced Lubrication and Sealing Materials,

School of Materials Science and Engineering,

Northwestern Polytechnical University,

Xi'an, Shaanxi 710072, China.

Email: tengfei.cao@nwpu.edu.cn

Q. Huang, X. Zhang, S. Wen, T. Cao

Department of Materials Science and Engineering,

Northwestern Polytechnical University,

Xi’an 710072, China.

Email: wenshifeng@nwpu.edu.cn

Y. Sun, Z. Wen

College of Electronics and Information

Shandong Key Laboratory of Micro-nano Packaging and System Integration,

Qingdao University,

Qingdao 266071, China.

Email: zwen@qdu.edu.cn

S. Lv, Qi Hu, L. Liu

School of Physics,

Beihang University,

Beijing 100191, China.

Jingsheng Chen, S. Shi

Department of Materials Science and Engineering,

National University of Singapore,

117575, Singapore


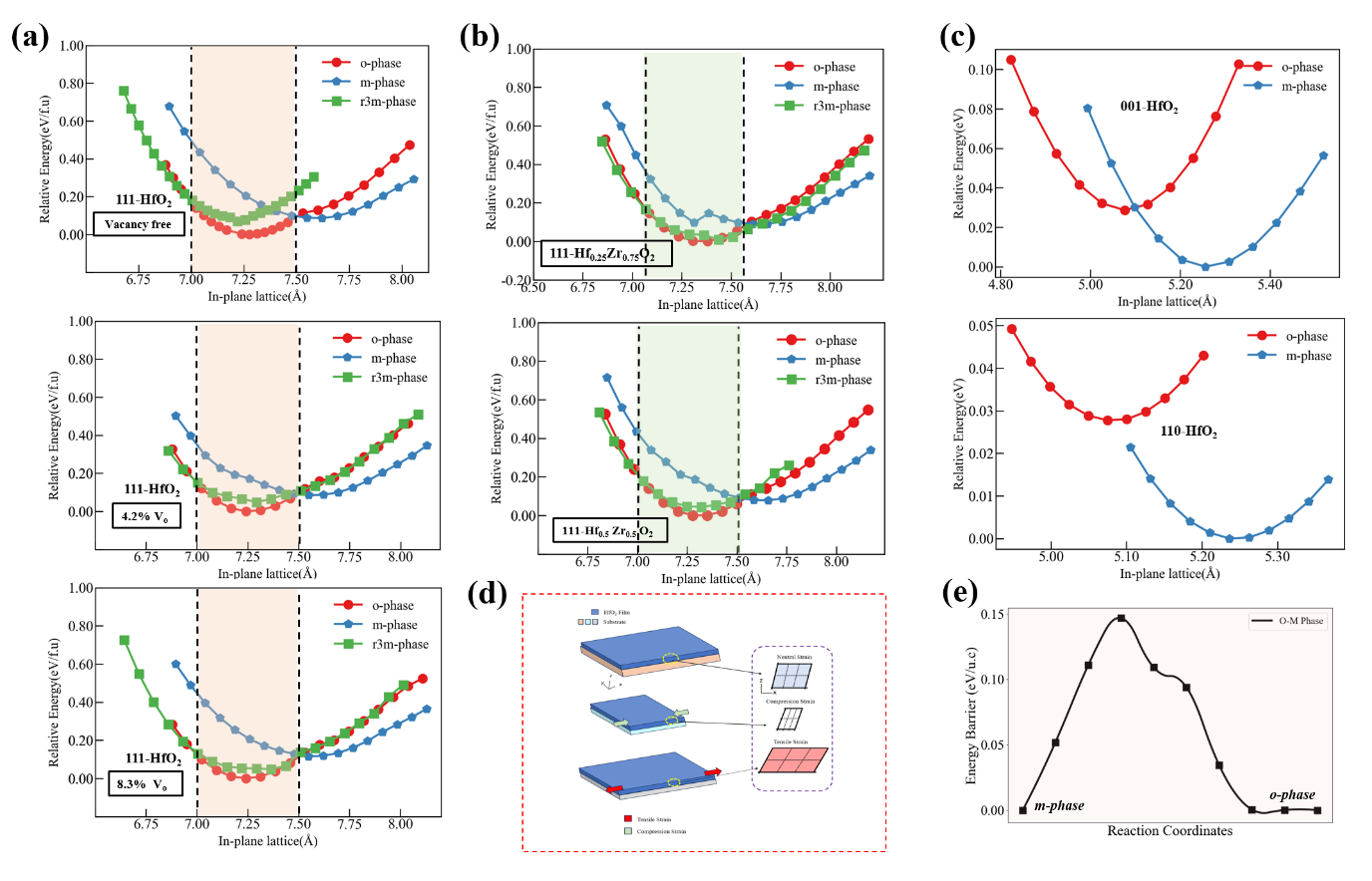


**Figure S1.** Stability of HfO_2_ films in (111), (001), and (110) orientations under different confined epitaxial strain (a) The energy curves of (111) oriented HfO_2_ *o-, m- and r3m* phases with respect to in-plane lattices under different oxygen vacancy concentrations, where the colored regions show the in-plane lattice stability window for the *o*-phase. (b) Phase stabilities of HZO films under different Hf and Zr alloying ratios of *o-, m- and r3m*-phase under different epitaxial strain. (c) Phase stabilities of *o-* and *m*-phase in (001) and (110) orientations under epitaxial strain. (d) Schematic diagram of HfO_2_ films on substrate under different epitaxial strain condition. (e) The calculated *o*- to *m*-phase transition energy barrier.


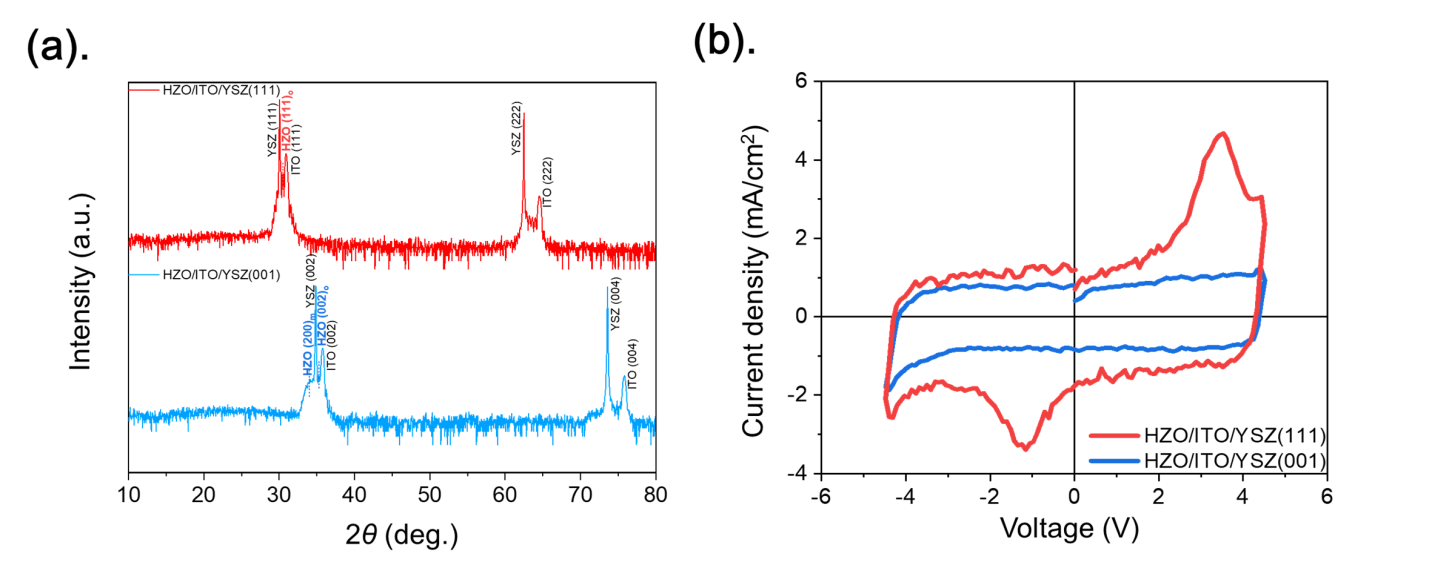


**Figure S2.** X-ray Diffraction patterns and polarization-switching currents (a) XRD patterns of the HZO thin films grown (111)- and (001)-oriented YSZ substrtates buffered with ITO bottom electrodes, showing presence of the monoclinic phase along the (001) direction compared to the (111) direction where only the ferroelectric orthorhombic phase is present. (b) Polarization-swtiching currents of the Pt/HZO/ITO thin-film capacitors fabricated on the (111)- and (001)-oriented YSZ substrates.


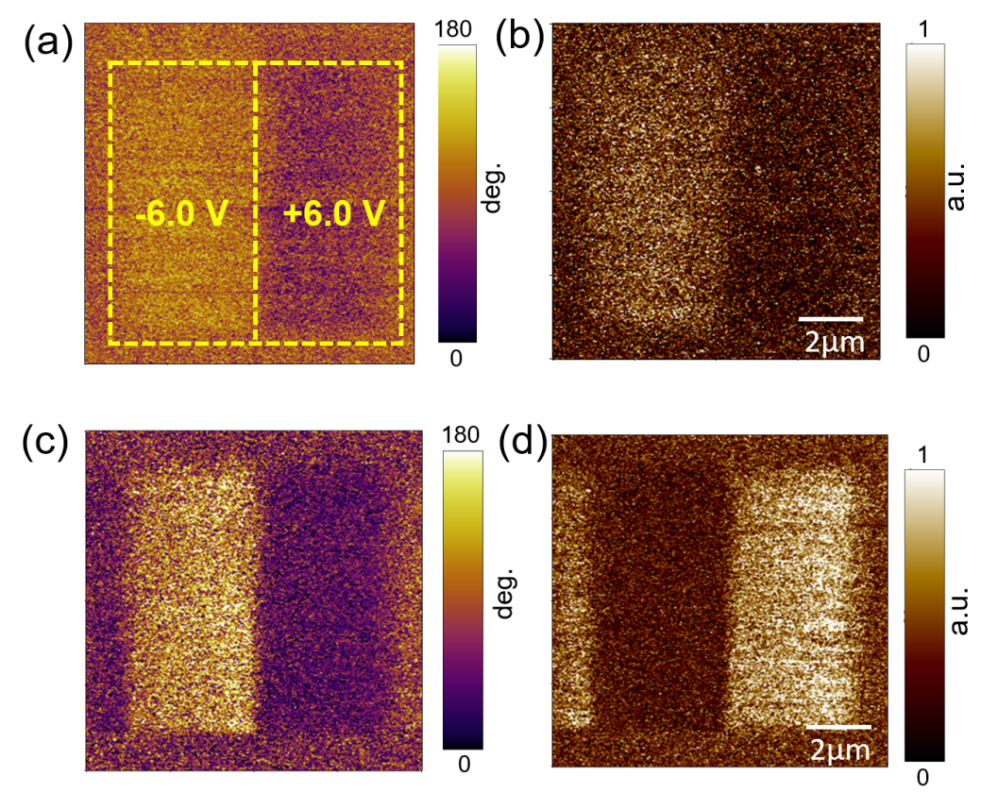


**Figure S3.** PFM phase (a, c) and amplitude (b, d) images of the HZO/ITO/YSZ (001) (a, b) and the HZO/ITO/YSZ (111) (c, d) heterostructures, respectively, collected after the tip-poling by +/-6.0 V according to the yellow boxes in (a). It is clear that the 7 nm-thick HZO along (111) direction exhibits pronounced piezoresponse contrast after the antiparallel poling, suggesting the robust ferroelectric polarizations. However, in the HZO along the (001) direction, there are almost no changes in both the phase and amplitude signals.


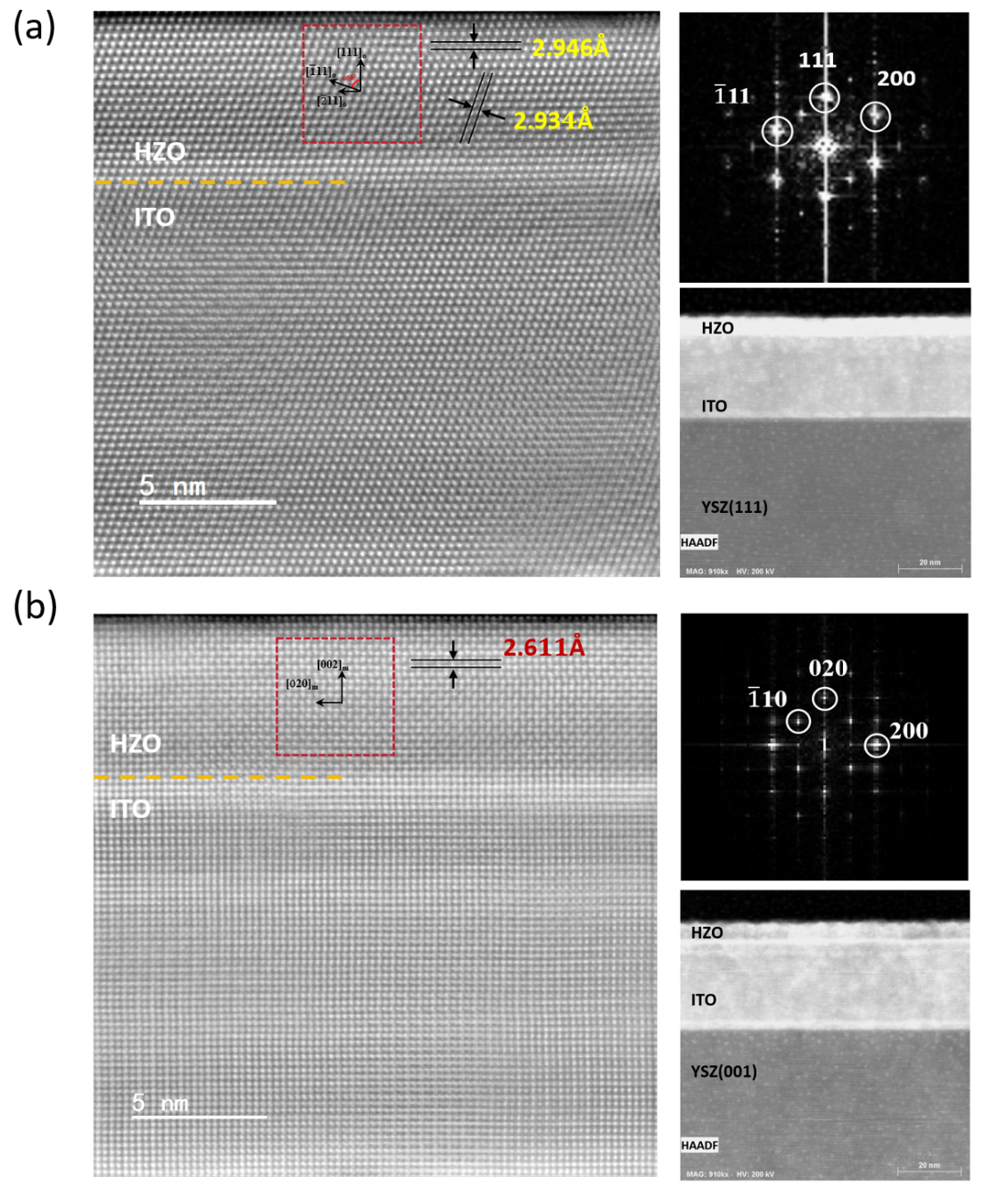


**Figure S4.** (a,b) HAADF-STEM image of the HZO/ITO/YSZ 5nm thin-film heterostructures, magnified HAADF-STEM image and corresponding fast fourier transform (FFT) pattern of the HZO(111) and HZO(001) layer indicated by the dashed red box in (a), for phase analysis.


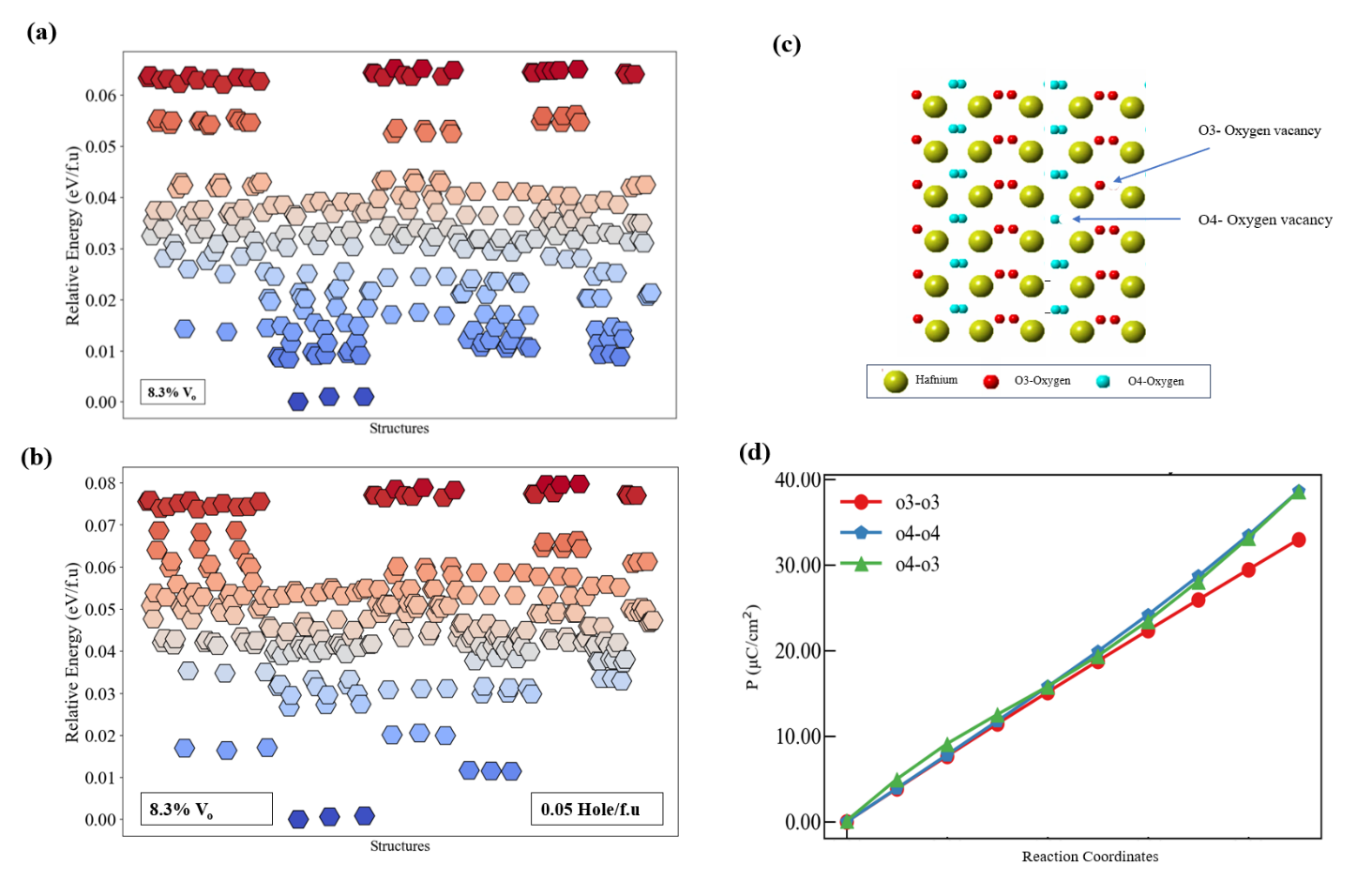


**Figure S5.** DFT Calculated Energies of (111) Ferroelectric HfO_2_ (a) Relative energies of 8.3% oxygen vacancy structures generated by creating all 276 oxygen vacancy configurations. (b) Relative energies of 276 structures, each with 8.3% oxygen vacancy and under 0.05 Hole/f.u. doping. The magnitude of the energies from low to high are indicated with a blue to red color gradients, respectively. (c) Illustrative diagram of O3 and O4 oxygen vacancy. (d) Berry Phase calculated polarization of (111)-HfO_2_ with different oxygen vacancy sites (O3-O4, O3-O3, O4-O4).

|  |  | In-plane Lattice |  |
| --- | --- | --- | --- |
| Strain | m-phase(Å) | o-phase(Å) | r3m-phase(Å) |
| -5% | 6.89 | 6.88 | 6.86 |
| -4% | 6.97 | 6.95 | 6.93 |
| -3% | 7.04 | 7.02 | 7.00 |
| -2% | 7.11 | 7.09 | 7.08 |
| -1% | 7.19 | 7.17 | 7.15 |
| 0% | 7.26 | 7.24 | 7.22 |
| 1% | 7.33 | 7.31 | 7.29 |
| 2% | 7.40 | 7.38 | 7.36 |
| 3% | 7.48 | 7.42 | 7.43 |
| 4% | 7.55 | 7.45 | 7.51 |
| 5% | 7.62 | 7.53 | 7.58 |

**Table S1**. In-plane lattices and the related epitaxial strain for (111) oriented m-, o- and r3m-phase

.

**Table S2**. All the substrates that support the growth of o-phase r3 strained (111) HfO₂, classified by category, and including their net type, in-plane lattice parameter, and lattice mismatch sourced from the material project database. Also, to stabilize the (111)-oriented orthorhombic HfO₂ phase with in-plane window is **7.0–7.5 Å**, the match alone isn’t everything, chemistry, surface symmetry, thermal expansion, and other factors account for the success. Here we recommend a lattice mismatch ≤ ≈ 7% as a more favourable substrate.

| **Substrate** | **Surface Type** | **In-plane Lattice (Å)** | **Mismatch** | **Category** |
| --- | --- | --- | --- | --- |
| YSZ (111) | Triangular | 7.27 | –0.95% | Fluorite oxide |
| CeO₂ (111) | Triangular | 7.65 | +4.22% | Metal oxide |
| MgO (111) | Rocksalt (111) | 7.29 | –0.68% | Rocksalt oxide |
| In₂O₃ (111) | Triangular | 7.16 | –2.5% | Metal oxide |
| Gd₂O₃ (111) | Triangular | 7.65 | +4.2% | Metal oxide |
| Sc₂O₃ (111) | Triangular | 6.99 | –4.9% | Metal oxide |
| ZrO₂ (111, cubic) | Fluorite (111) | 7.20 | –1.91% | Fluorite oxide |
| TiO₂ (110) | Rutile (110) net | 6.53 – 7.21 | –1.8% to –11% | Rutile oxide |
| Al₂O₃ (0001) | Corundum (0001) | 9.52 | +29.7% | Corundum oxide |
| LaAlO₃ (001) | Perovskite (001) net | 7.58 | +3.24% | Perovskite |
| CaTiO₃ (001) | Perovskite (001) net | 7.64 | +4.09% | Perovskite |
| NdGaO₃ (001) | Perovskite (001) net | 7.72 | +5.18% | Perovskite |
| LaGaO₃ (001) | Perovskite (001) net | 7.78 | +5.99% | Perovskite |
| LSAT (001) | Perovskite (001) net | 7.74 | +5.40% | Perovskite |
| LSMO (001) | Perovskite (001) net | 7.74 | +5.45% | Perovskite |
| SrTiO₃ (001) | Perovskite (001) net | 7.81 | +6.40% | Perovskite |
| SrRuO₃ (001) | Perovskite (001) net | 7.86 | +7.08% | Perovskite |
| DyScO₃ (001) | Perovskite (001) net | 7.89 | +7.44% | Perovskite |
| GdScO₃ (001) | Perovskite (001) net | 7.94 | +8.12% | Perovskite |
| TbScO₃ (001) | Perovskite (001) net | 7.92 | +7.90% | Perovskite |
| KTaO₃ (001) | Perovskite (001) net | 7.98 | +8.72% | Perovskite |
| BaTiO₃ (001) | Perovskite (001) net | 7.98 | +8.72% | Perovskite |
| PbTiO₃ (001) | Perovskite (001) net | 7.94 | +8.19% | Perovskite |
| BiFeO₃ (001) | Perovskite (001) net | 7.92 | +7.90% | Perovskite |
| SrSnO₃ (001) | Perovskite (001) net | 8.08 | +10.1% | Perovskite |
| SmScO₃ (001) | Perovskite (001) net | 7.89 | +7.37% | Perovskite |
| NdScO₃ (001) | Perovskite (001) net | 7.90 | +7.64% | Perovskite |
| PrScO₃ (001) | Perovskite (001) net | 7.86 | +7.08% | Perovskite |


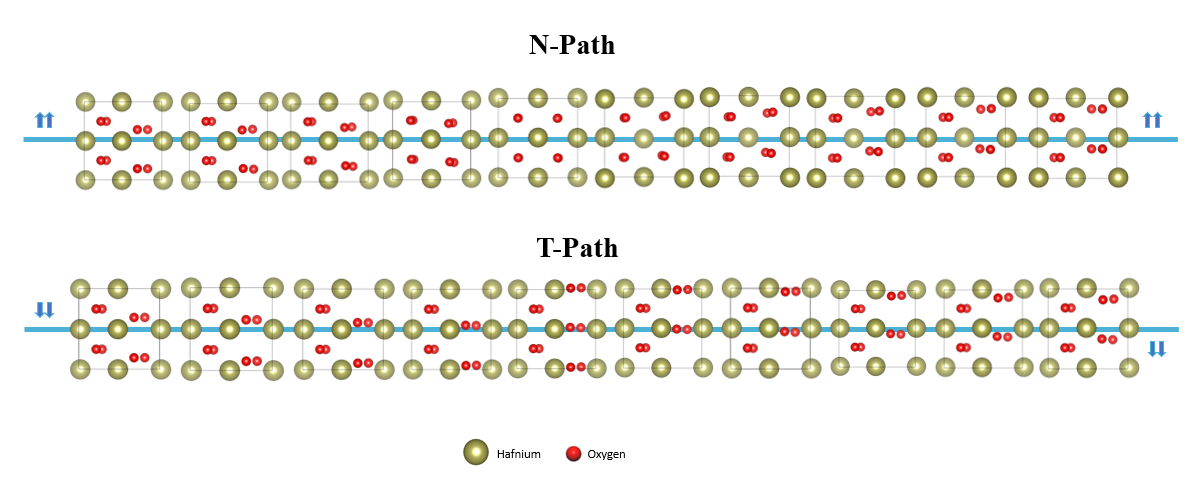


**Figure S6**. Atomic structures of HfO_2_ for both N- and T-path 180° polarization switching. Initial, intermediate, and final atomic structures from polar up to polar down. The blue line indicates the Hf-Hf plane, and the arrows shows polar oxygen atom movement.


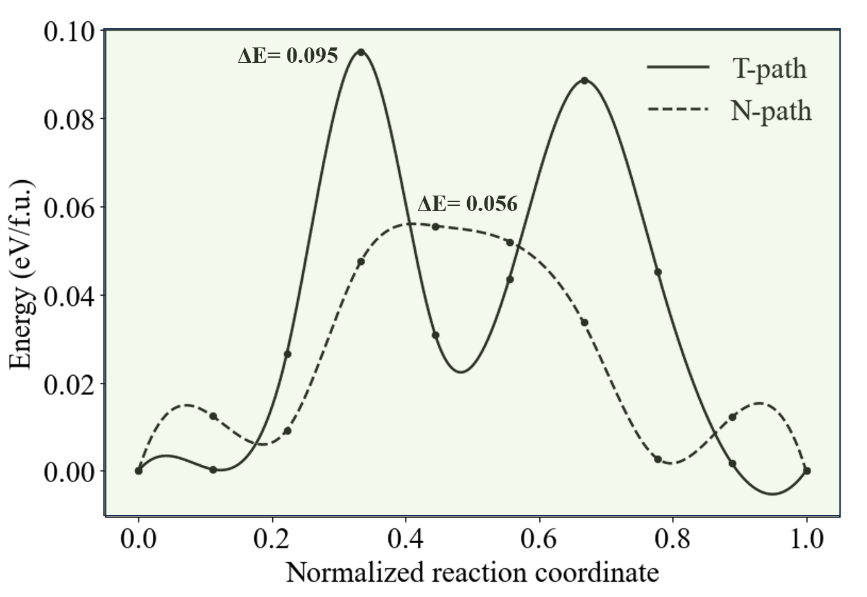


**Figure S7.** VCNEB energy barrier of N- and T-path polarization switching of 12-atom orthorhombic unit cell of ferroelectric HfO₂ oriented along the [001] direction, which corresponds to the symmetry-allowed polar axis and the experimentally relevant out-of-plane orientation in thin films.


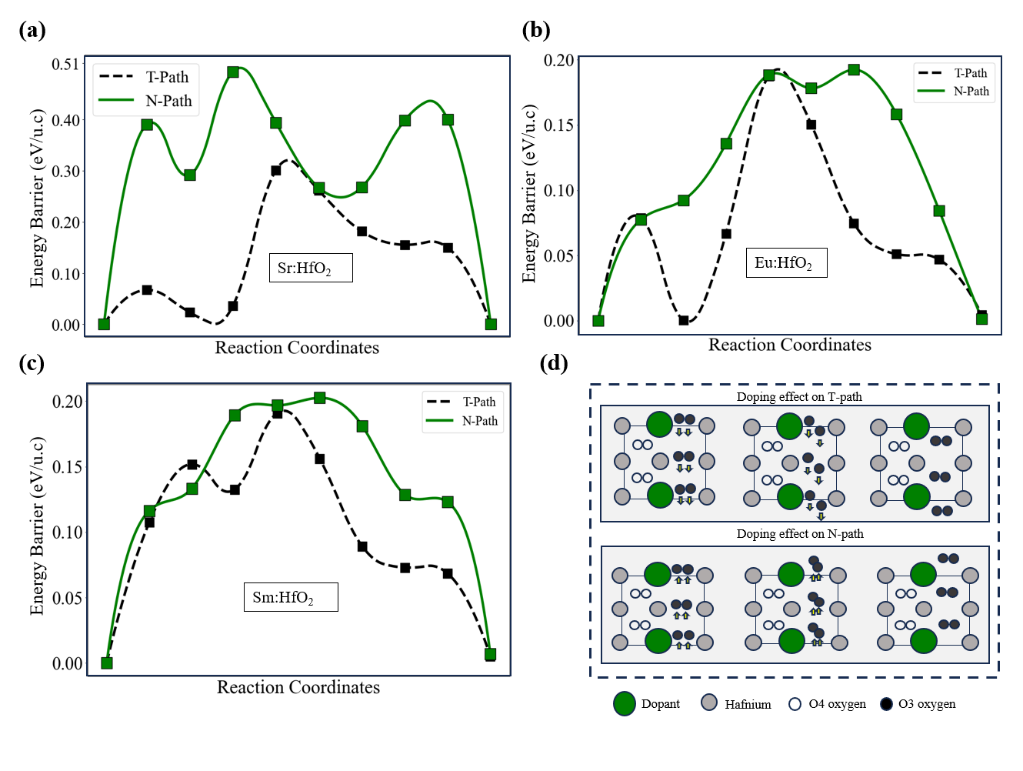


**Figure S8.** Cation Doping Effects on Switching Barriers of N-path and T-path for Ferroelectric HfO2. Polarization switching (polar up to polar down) energy profile for doped HfO2 N-path and T-path with 25% concentration of (a)Sr, (b) Sm and (c) Eu dopants. (d) Schematic structure deformations in cation doped HfO_2_ for N-path and T-path.


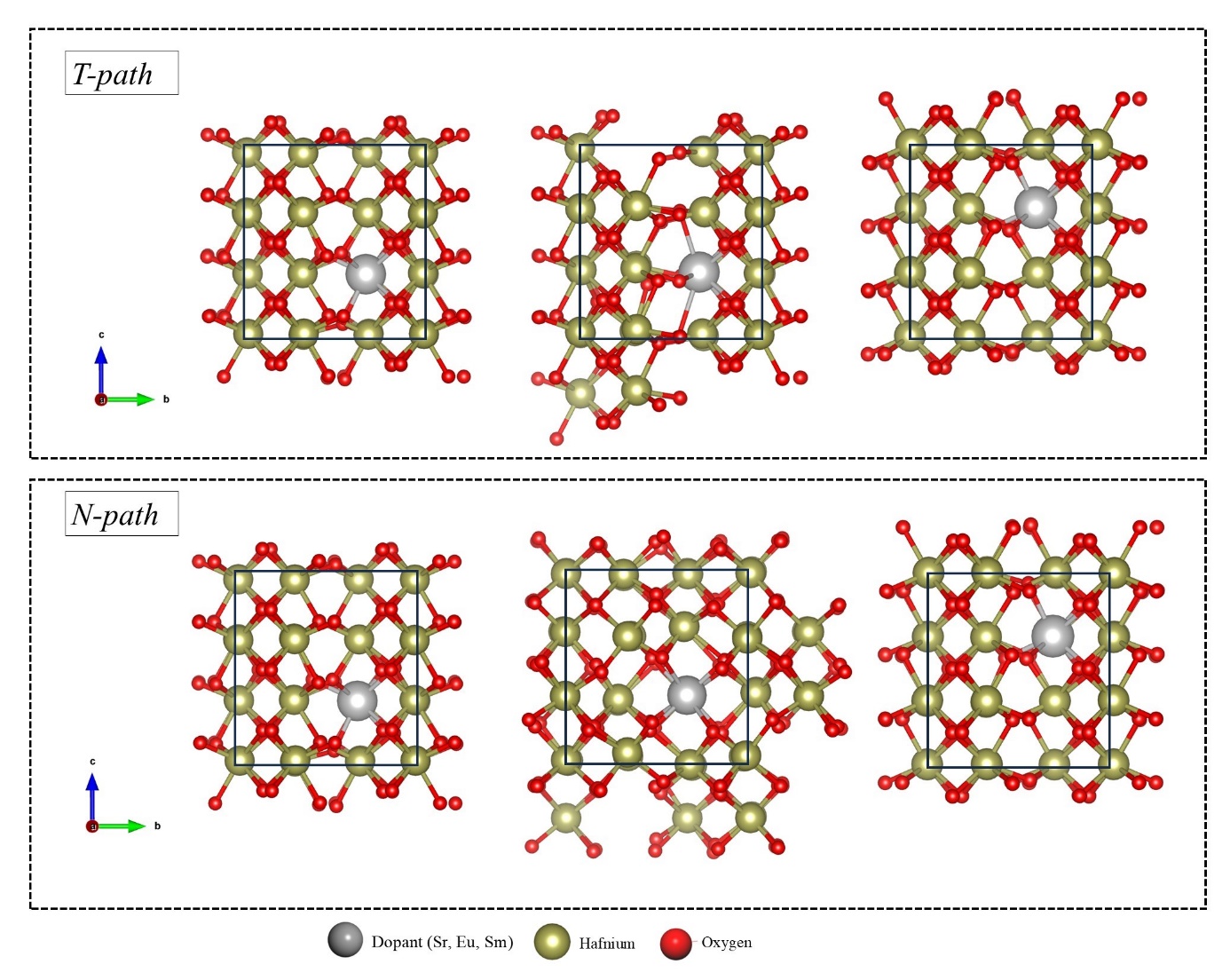


**Figure S9.** Optimized atomic structures for initial, intermediate and final images in ferroelectric switching paths in doped (3.125%) HfO_2_ cross (T-path) and non-cross (N-path) pathways.


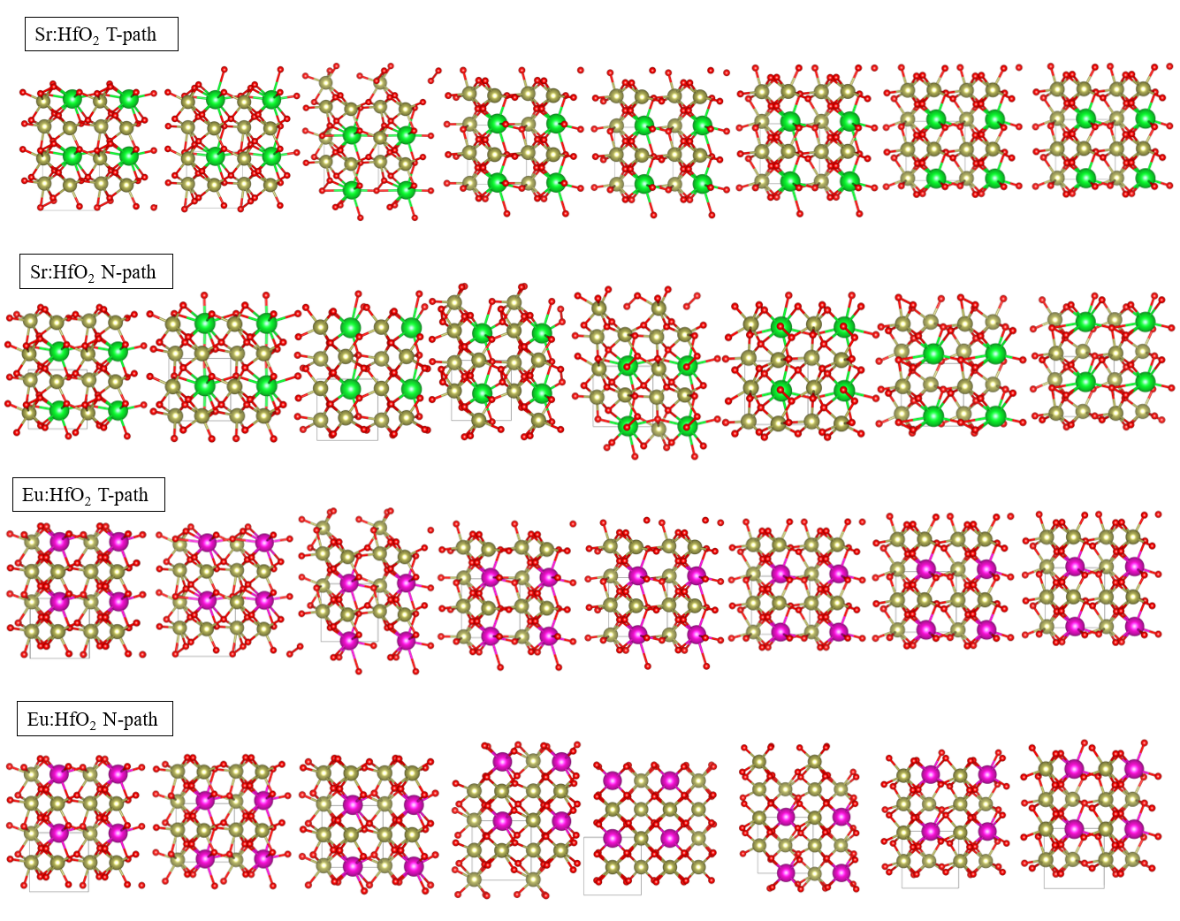


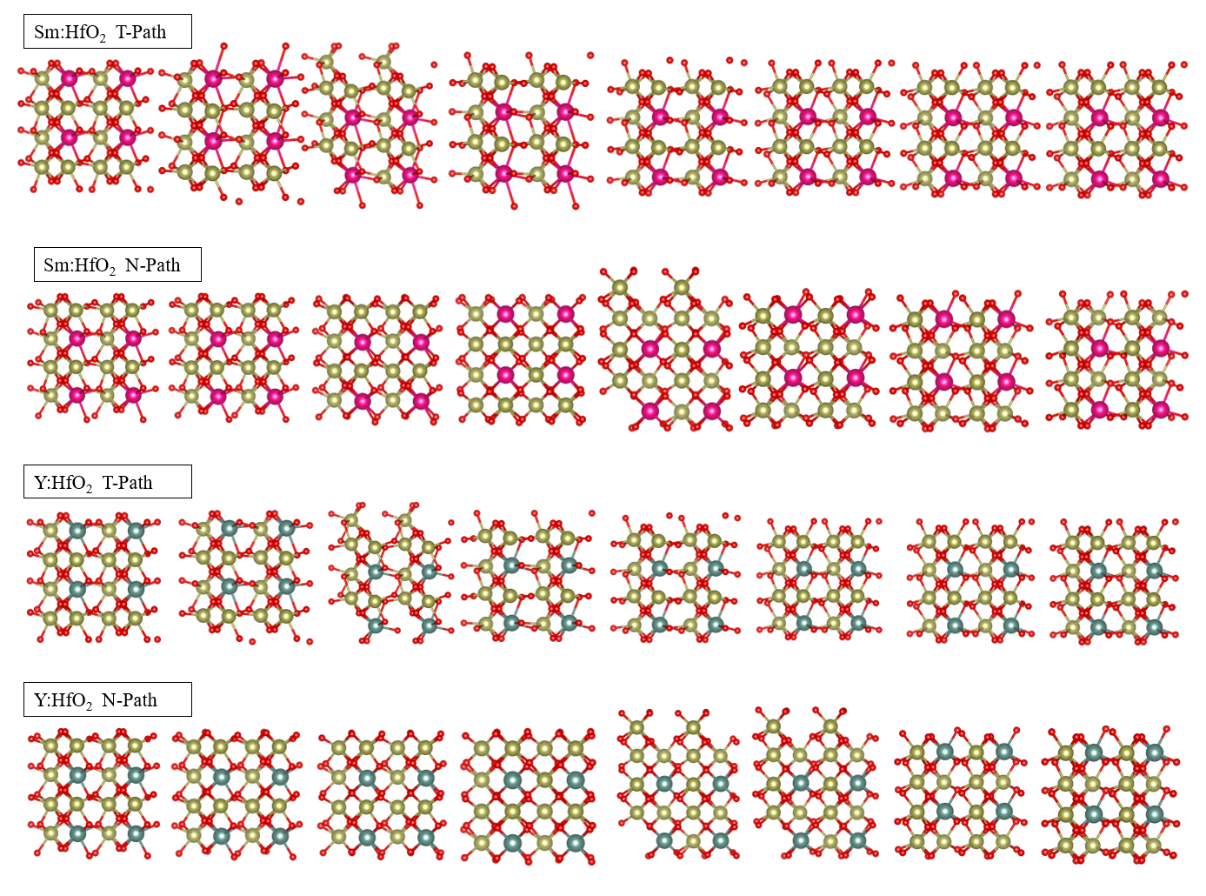


**Figure S10.** Atomic structures for all images in ferroelectric switching paths in Sr, Eu, Sm and Y doped HfO_2_ cross (T-path) and non-cross (N-path) pathways.


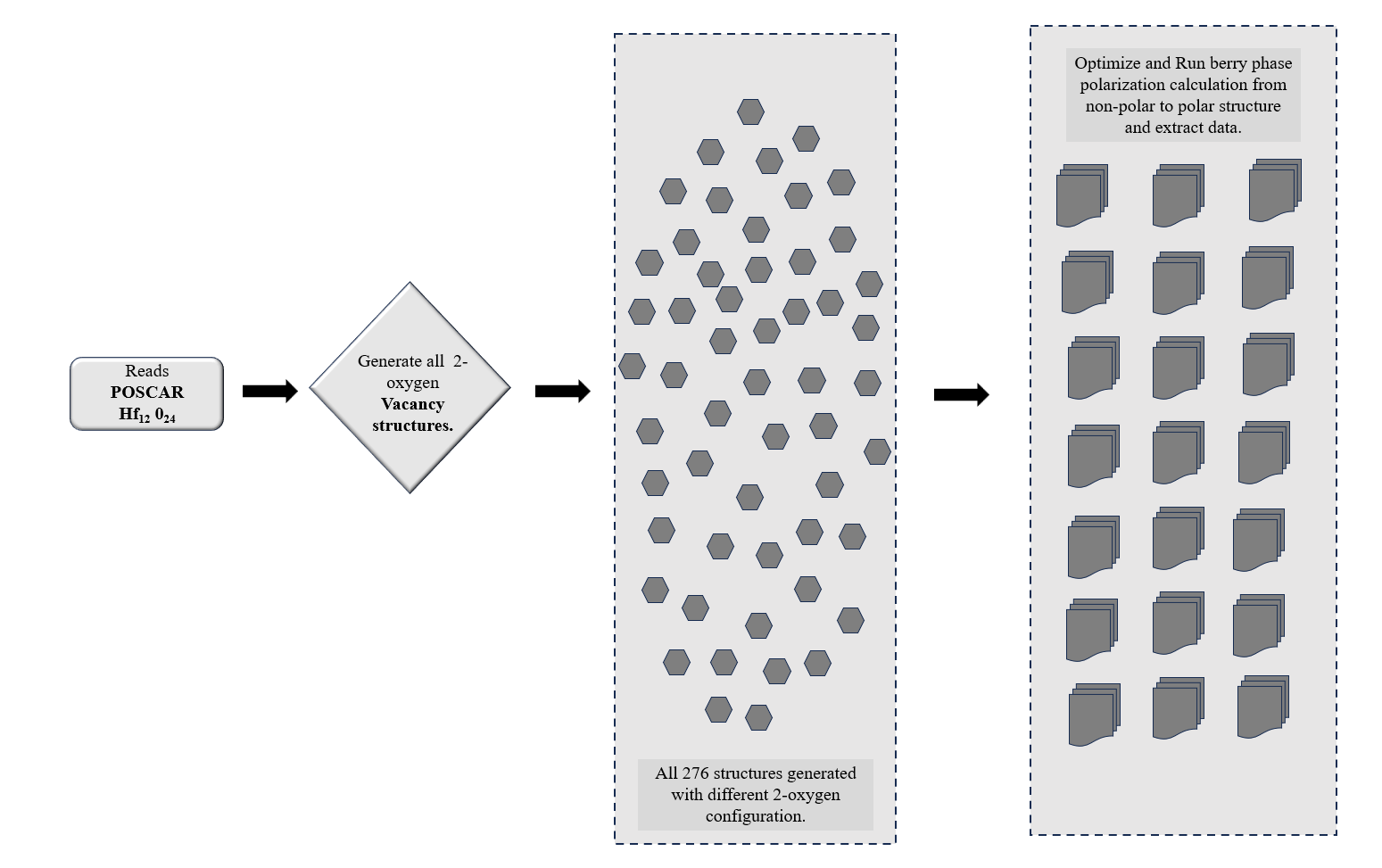


**Figure S11**: Workflow for generating and evaluating oxygen-vacancy configurations and their polarization response. Starting from the POSCAR of HfO₂, all symmetrically distinct two–oxygen-vacancy structures are generated (276 configurations in total). Each configuration is then structurally optimized, followed by Berry-phase polarization calculations along the nonpolar-to-polar transformation path to extract polarization data.


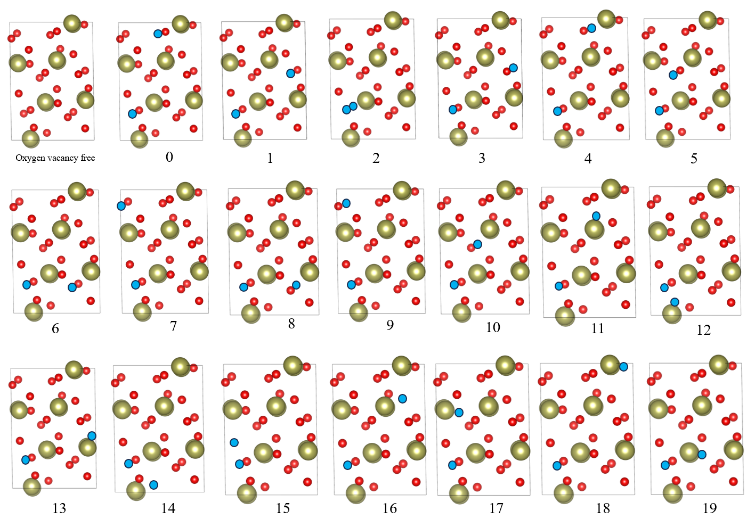

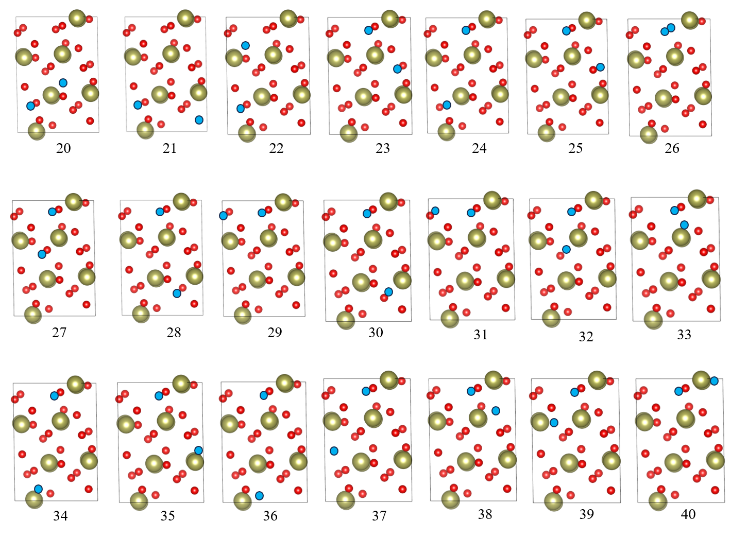

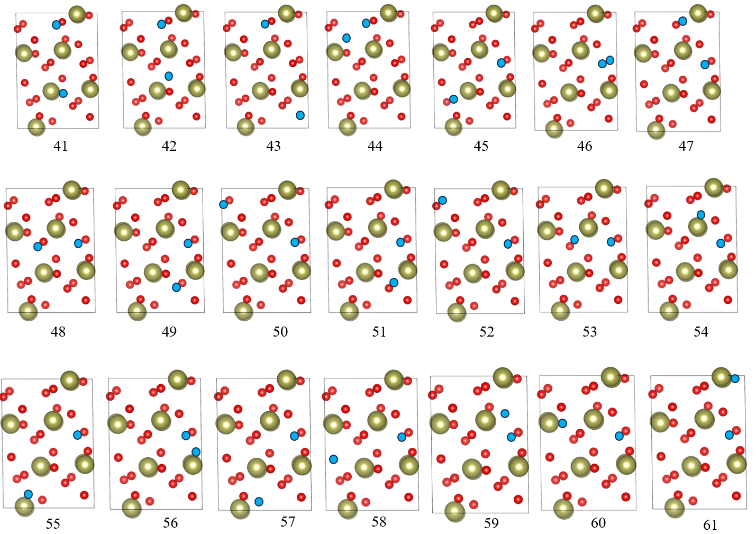

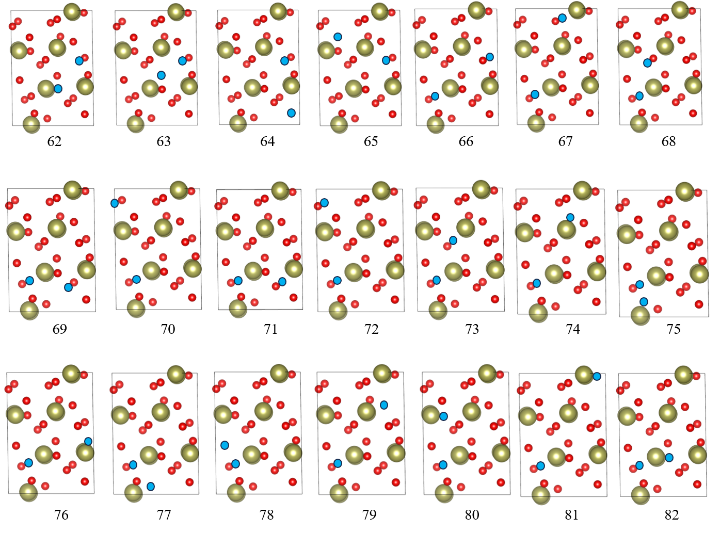


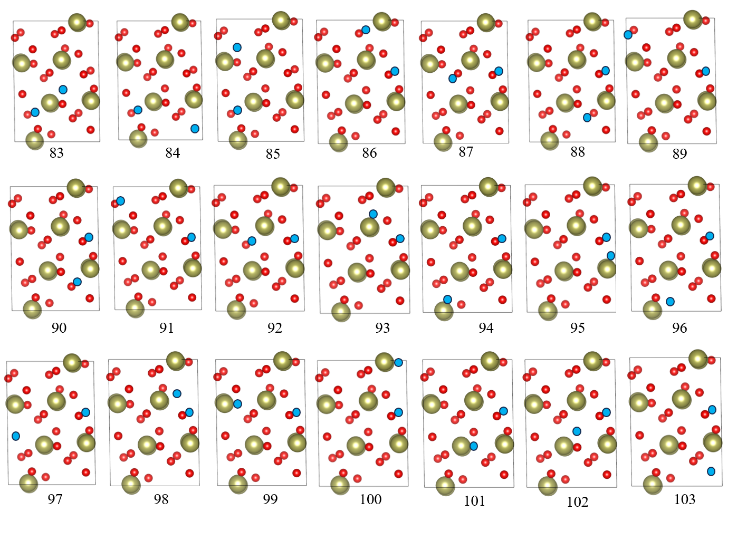

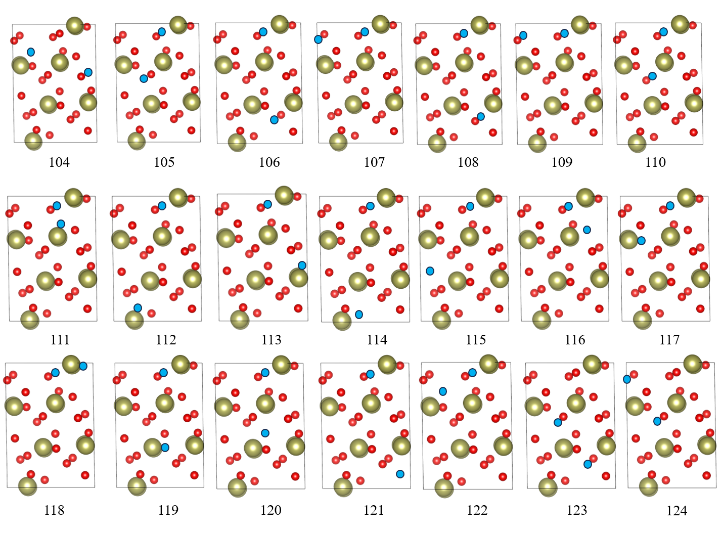


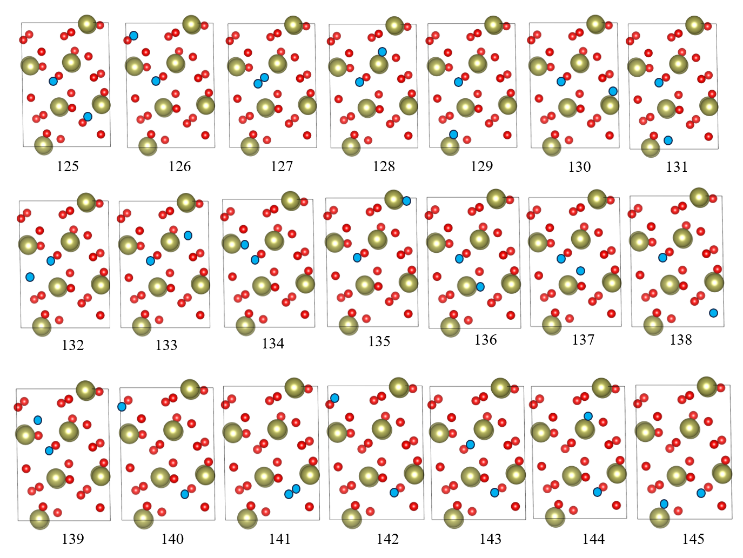

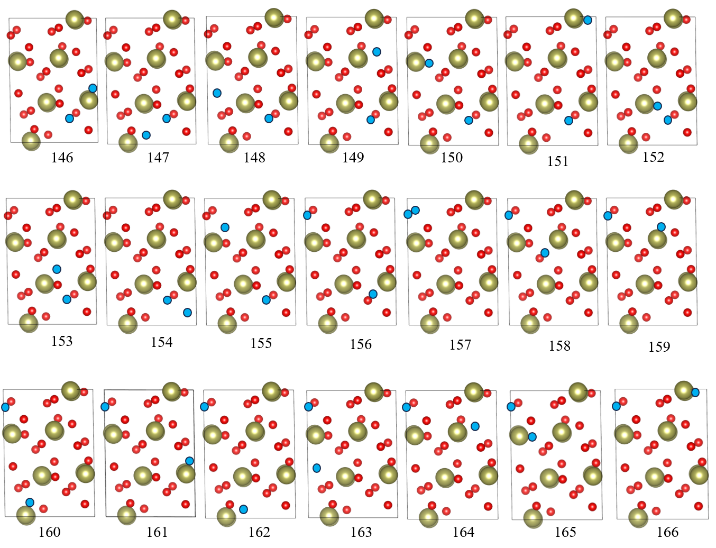

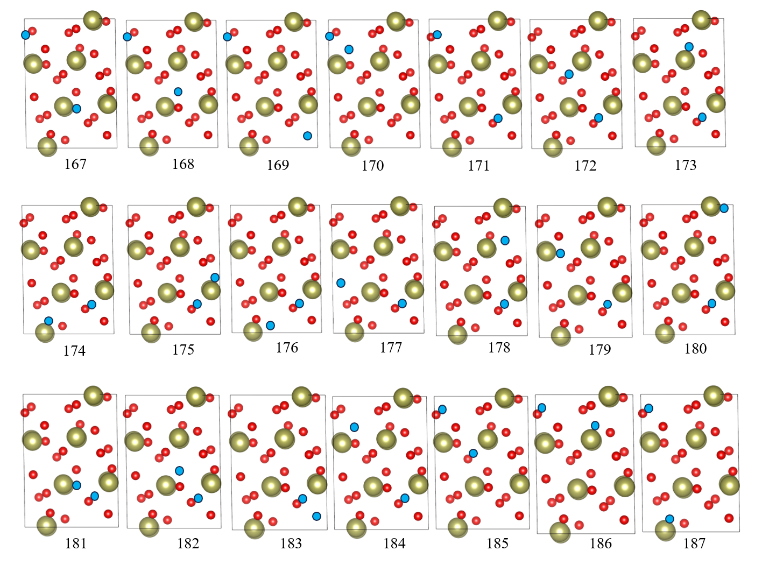

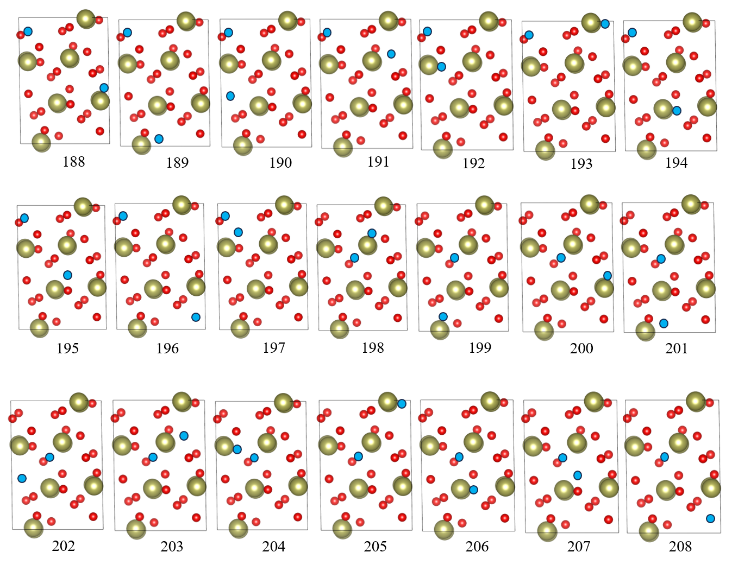


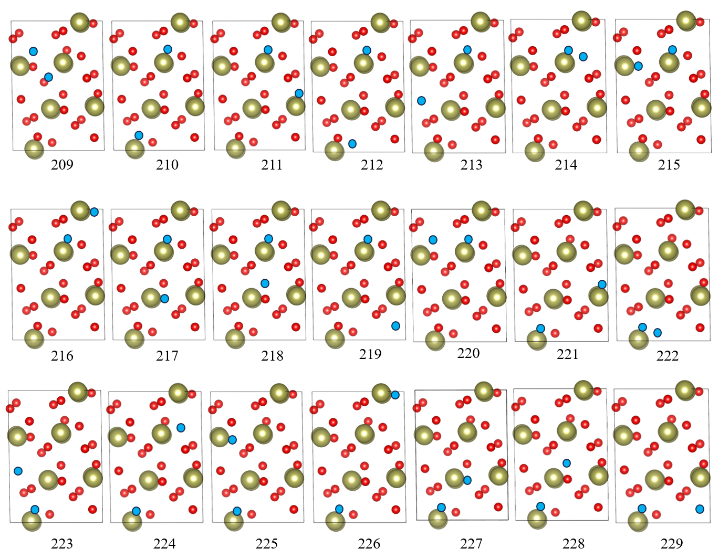

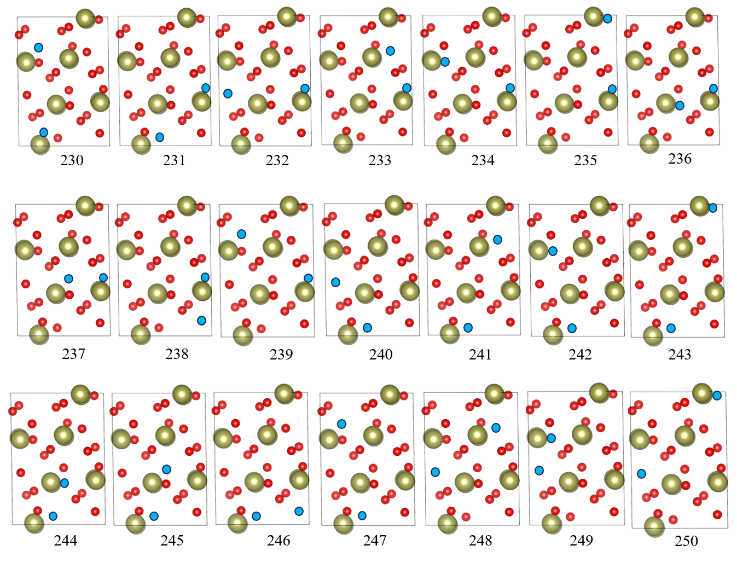

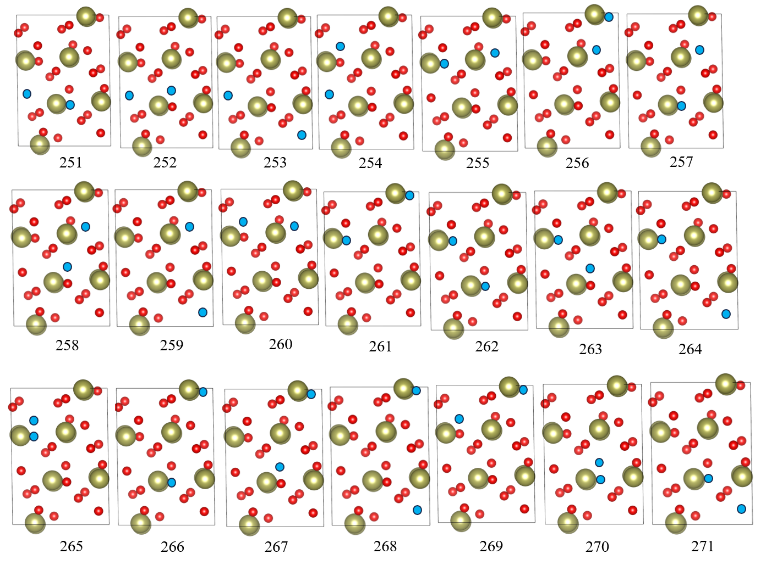

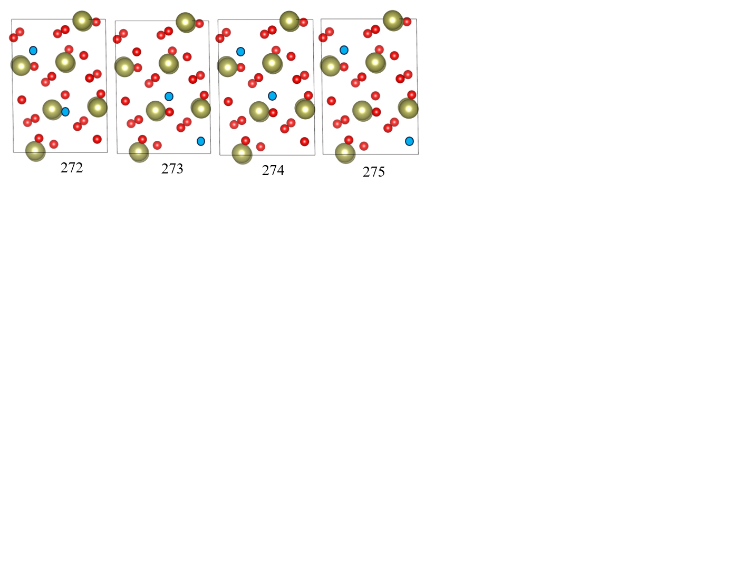


**Figure S12.** **Atomic structures of HfO_2_ ferroelectric phases under oxygen vacancy**: All 8.3% oxygen vacancy structures generated; 276 vacancy sites variation.
